# Piezo1 Mechanotransduction in Skeletal Muscle: Convergence with Noncoding RNA Regulation in Myogenesis, Regeneration, and Sarcopenia

**DOI:** 10.3390/ijms27167084

**Published:** 2026-08-07

**Authors:** Thanh Huu Phan Ngo, Jiwon Oh, Hyeong Jun Kim, Wan Lee

**Affiliations:** 1 Department of Biochemistry, Dongguk University College of Medicine, 123 Dongdae-ro, Gyeongju 38066, Republic of Korea; thngo139@gmail.com (T.H.P.N.); jiwon8757@naver.com (J.O.); bdbd147@naver.com (H.J.K.); 2 Section of Molecular and Cellular Medicine, Medical Institute of Dongguk University, Gyeongju 38066, Republic of Korea

**Keywords:** Piezo1, mechanotransduction, skeletal muscle, satellite cells, myoblast fusion, noncoding RNA, microRNA, MRTFA-SRF, YAP/TAZ, sarcopenia

## Abstract

Skeletal muscle is a continuously load-bearing tissue whose growth, repair, and age-related decline are governed by mechanical signals; failure of this mechano-regulation underlies disuse atrophy and sarcopenia. Piezo1, a mechanically activated cation channel, has emerged as a tractable transducer of these signals in muscle, contributing to satellite-cell quiescence and senescence, regenerative division, myoblast fusion, and the response to loading and unloading. In parallel, the myogenic noncoding RNA program is among the best defined in any lineage, with myomiRs miR-1/133/206, the long noncoding RNA LINC-MD1, and the circular RNA circ-ZNF609 being established regulators of the proliferation-to-differentiation transition. These layers are linked because Piezo1-evoked calcium influx feeds the RhoA/ROCK-actin-MRTFA-SRF and YAP/TAZ axis that drives myogenic transcription, yet no direct coupling between Piezo1 and noncoding RNAs has been demonstrated in skeletal myocytes. Drawing on validated precedents from vascular, cardiac, and tendon tissues, this review consolidates the two pillars, frames their convergence as a testable question, distinguishes validated relationships from hypotheses, and proposes three falsifiable predictions using an unbiased candidate selection strategy. The contribution of this review is this testable framework rather than any specific candidate list. Mechanically tunable noncoding RNAs may thus represent an underexplored node for counteracting disuse atrophy and sarcopenia.

## 1. Introduction

Skeletal muscle is a mechanically active organ throughout life. It generates force, bears load, and continuously experiences stretch, shear, and changes in substrate stiffness, adapting its mass and composition to these mechanical demands: chronic loading promotes hypertrophy, whereas unloading, immobilization, and inactivity drive atrophy. The clinical consequences of failed mechano-adaptation are substantial. Sarcopenia, the age-related loss of muscle mass and function, is now formally recognized as a muscle disease with defined diagnostic criteria centered on low muscle strength and is a major contributor to frailty, falls, fractures, and loss of independence in older adults [1]. Its pathophysiology is multifactorial, spanning motor-unit remodeling, mitochondrial dysfunction, anabolic resistance, chronic low-grade inflammation, and a declining capacity of satellite cells to support repair [2]. Despite this clinical importance, there are, at present, no widely accepted pharmacological therapies for muscle atrophy and wasting [3], leaving resistance exercise, an inherently mechanical stimulus, as the principal effective countermeasure. Understanding how muscle cells sense and respond to mechanical cues is, therefore, both a fundamental question in cell biology and a prerequisite for rational intervention in muscle wasting.

Central to this question is the identification of the molecular sensors that convert mechanical force into biochemical signals. Among these, the mechanically activated cation channel Piezo1 has become a focal point. Throughout this review, we use mechanosensing to denote the detection of a mechanical stimulus and mechanotransduction to denote its conversion into a biochemical and transcriptional response. Piezo1 was identified as a pore-forming component of a distinct class of mechanically activated channels [4,5], and subsequent structural studies have revealed how its unusual architecture enables it to detect membrane tension and gate a cation-conducting pore [6,7,8,9]. Activation of Piezo1 admits calcium into the cell [10], placing Piezo1 upstream of a wide range of calcium-dependent signaling events. Although first characterized in non-muscle systems such as the vascular endothelium [10,11], Piezo1 is now recognized as a key mechanosensor in skeletal muscle itself.

Running in parallel with the study of mechanosensitive channels, noncoding RNAs (ncRNAs), comprising microRNAs (miRNAs), long noncoding RNAs (lncRNAs), and circular RNAs (circRNAs), have been established as central regulators of myogenesis. Unlike protein-coding transcripts, ncRNAs act largely through post-transcriptional mechanisms, fine-tuning the amplitude, timing, and reversibility of the differentiation program. The muscle-enriched myomiRs, along with a growing set of muscle lncRNAs and circRNAs, collectively sharpen the transition between proliferation and differentiation [12,13,14,15]. For much of the past two decades, the mechanotransduction and ncRNA fields advanced largely independently of one another.

That separation is now being bridged. In several mechanically loaded tissues outside skeletal muscle, Piezo1 has been shown to sit within bidirectional ncRNA circuits, acting both upstream of microRNAs that it induces and downstream of microRNAs that target it [16,17,18]. These precedents raise a question that has not yet been addressed in skeletal muscle: are Piezo1 signaling and the myogenic ncRNA program mechanistically coupled, and if so, with what consequences for differentiation, regeneration, and atrophy?

**Figure 1:**
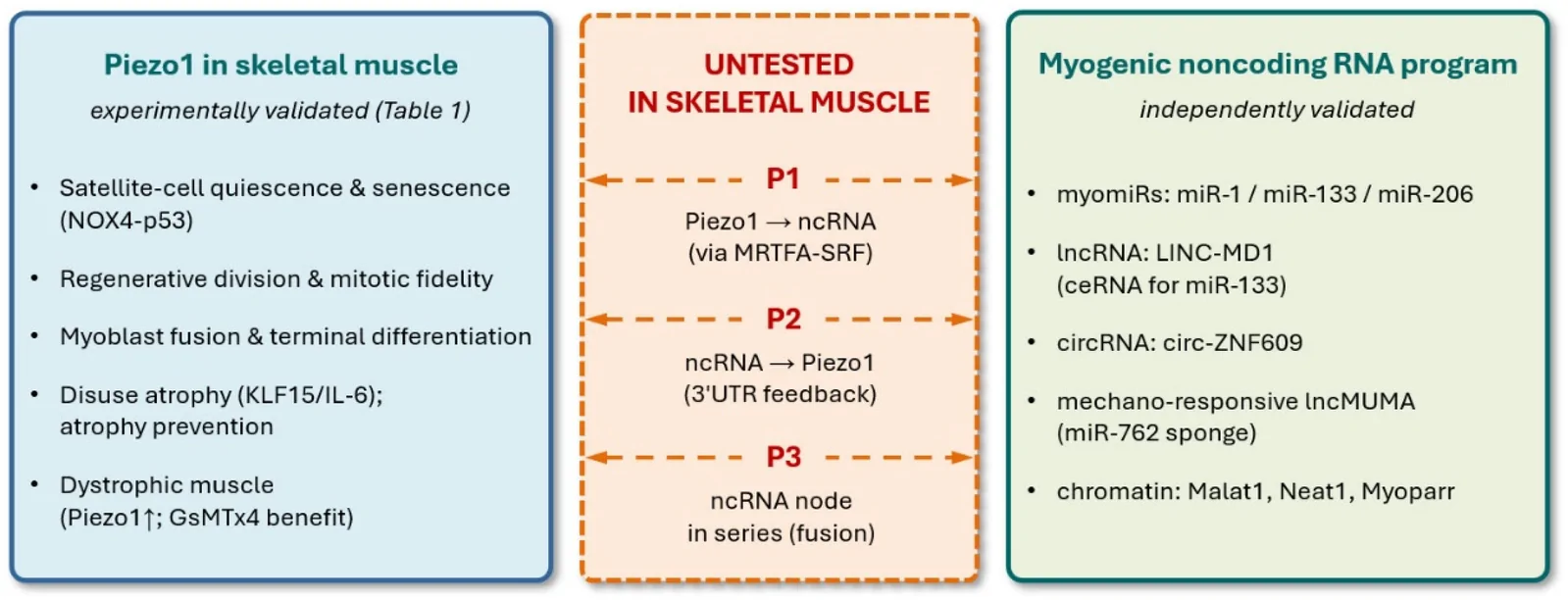
The Piezo1-noncoding RNA convergence gap in skeletal muscle: validated versus unexplored. Two validated pillars, the experimentally established Piezo1 functions in skeletal muscle (Table 1) and the independently validated myogenic noncoding RNA program, are separated by the untested coupling that this review examines (dashed, Predictions P1–P3).

This review is organized around that question. We first summarize the essential molecular features of Piezo1 (Section 2) and the now-substantial evidence for its roles in skeletal muscle (Section 3, with a synthesis in Table 1). We then trace the signaling route from Piezo1-evoked calcium to the myogenic transcriptional program through the actin-MRTFA-SRF and YAP/TAZ axes (Section 4) and review the myogenic ncRNA program that this machinery is positioned to regulate (Section 5). Section 6 directly confronts the central gap, drawing on validated precedents from other lineages and proposing three falsifiable predictions (Table 2), along with an unbiased strategy for candidate selection (Figure 1). Section 7 and Section 8 consider the disease and therapeutic implications, as well as the principal limitations and open questions. Throughout this paper, we are explicit about which relationships are experimentally validated in skeletal muscle and which remain hypotheses, since conflating the two has been a recurrent weakness in this fast-moving area. What makes this question tractable rather than merely unexplored is that the actin-MRTFA-SRF and YAP/TAZ machinery that is capable of relaying a Piezo1 signal to noncoding RNA loci is already present in myocytes (Section 4). Whether mechanical input actually reaches that layer can therefore be posed as a falsifiable hypothesis rather than left as open-ended speculation. This is intended as a forward-looking review: it synthesizes the established literature and, on that basis, defines a small number of specific, testable questions, rather than advancing a fixed position.

## 2. Molecular and Cellular Function of Piezo1

### 2.1. Discovery and Structural Architecture

Piezo1 (originally Fam38A) was identified in an unbiased screen as a pore-forming subunit sufficient to confer mechanically activated cation currents [4,5]. It is a large homotrimer that assembles into a three-bladed, propeller-like structure resolved by cryo-electron microscopy [6,7,8]. Each curved blade bows the membrane into a dome, and a long intracellular beam couples the blades to a central pore, converting membrane deformation into pore opening [6,7,8]. This 38-helix nano-bowl undergoes large in-plane area expansion, thought to underlie the channel’s high mechanosensitivity [27,28,29]. By bowing the bilayer, Piezo1 stores membrane tension so that flattening of the dome under load gates the pore through the lever-like beam [9], tuning it to membrane mechanics rather than to a single ligand.

### 2.2. Gating, Conductance, and Downstream Signaling

Piezo1 is gated by lipid-bilayer tension itself: removing the cytoskeleton does not abolish mechanosensitivity, so the membrane is the primary force-transmitting element [30]. This places it within the “force-from-lipids” model shared with other mechanosensitive channels, though tethered and cytoskeletal elements can modulate its responses [31]. Piezo1 is one of several mammalian force-gated channels, alongside Piezo2, TREK/TRAAK, TMEM63/OSCA, and the TMC channels, each tuned to distinct contexts [32]. As a non-selective cation channel with appreciable calcium permeability, Piezo1 produces rapid calcium influx on mechanical stimulation, limited by intrinsic inactivation. This calcium engages calmodulin, calcineurin/NFAT, and Rho-family GTPase signaling, linking the channel to rapid cytoskeletal remodeling and slower transcription. In muscle, this calcium-to-cytoskeleton-to-transcription relay connects Piezo1 to the myogenic program (Section 4).

### 2.3. Tissue Distribution, Channelopathies, and Physiological Roles

Piezo1 is broadly expressed, and its non-muscle roles establish the general logic later applied to muscle. In the vascular endothelium, it transduces shear stress, and its loss disrupts vascular development and is embryonic-lethal in mice [10,11,33,34]. In larval zebrafish, body motion rather than heartbeat-driven flow is the dominant trigger of Piezo1-dependent endothelial calcium events, directly coupling muscle-generated movement to vascular mechano-signaling [35].

Human channelopathies show that Piezo1 function is physiologically decisive. Gain-of-function PIEZO1 mutations cause hereditary xerocytosis, the first link between a mechanosensitive channel and genetic disease [36]. Via the KCa3.1 Gardos channel, Piezo1 controls erythrocyte volume, thereby driving the dehydration observed in the disorder [37]. Loss-of-function mutations instead cause lymphatic dysplasia [33]. Both excessive and insufficient Piezo1 activity are therefore pathological, a theme that recurs in the dystrophic muscle data below.

Piezo1 also acts across the musculoskeletal system. In bone, it is required for the skeletal response to loading, and a Piezo1 agonist increases bone mass, identifying it as an anabolic target [38]. In articular cartilage, inflammatory signaling upregulates Piezo1 to drive a calcium-dependent, F-actin-rarefying feed-forward loop that aggravates osteoarthritis [39,40], a direct Piezo1-calcium-to-F-actin link of the kind invoked for muscle in Section 4. This breadth establishes Piezo1 as a general-purpose mechanotransducer whose calcium-to-cytoskeleton logic is expected to operate in muscle, while its wide, dose-sensitive expression complicates therapeutic targeting (Section 7).

### 2.4. Pharmacological Tools

Two tools recur below and have been essential for establishing causality. The small molecule Yoda1 is a selective Piezo1 agonist that lowers the activation threshold, mimicking mechanical stimulation [41], whereas the peptide GsMTx4 inhibits Piezo1 and related channels, blunting stretch-evoked cation flux [42]. The toolkit has since expanded to include the Yoda1 analog Dooku1, which reversibly antagonizes Yoda1-evoked activation without affecting constitutive activity [43]. With genetic gain- and loss-of-function approaches, these tools enable the causal tests in the next section, though Piezo1 pharmacology can differ across species and cell types [44].

## 3. Piezo1 in Skeletal Muscle Biology

**Figure 2:**
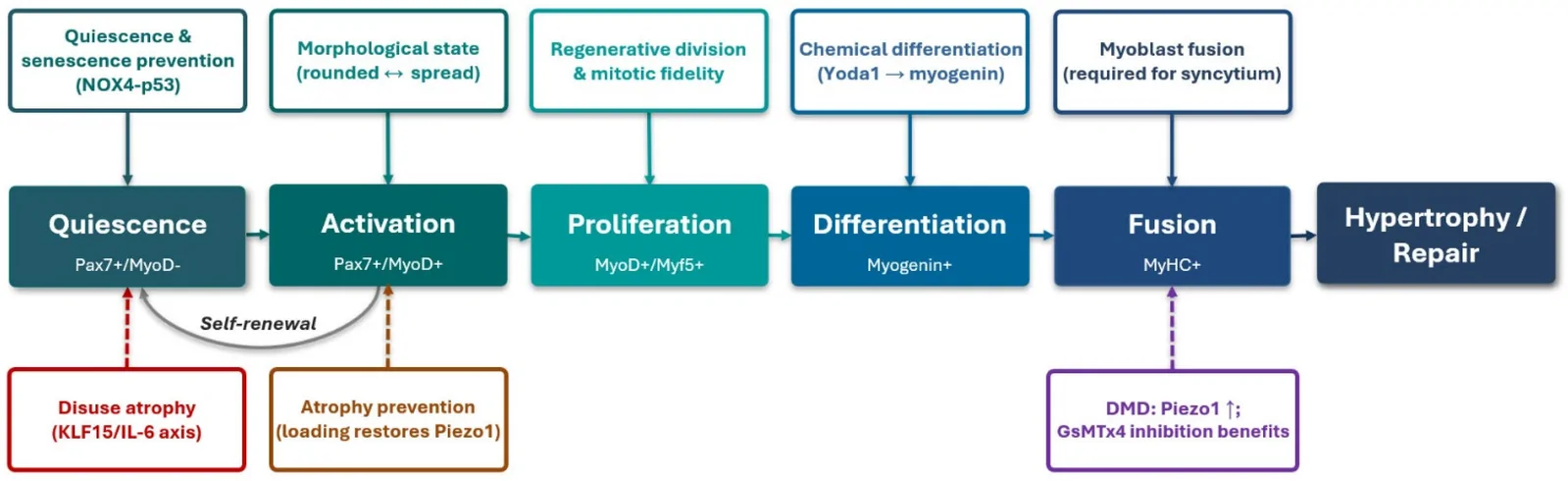
Satellite-cell lifecycle and the validated regulatory roles of Piezo1. The eight experimentally validated Piezo1 functions summarized in Table 1 are mapped onto satellite-cell progression from quiescence through activation, proliferation, differentiation, and fusion to hypertrophy and repair, with the self-renewal route indicated.

Work over the past five years has placed Piezo1 at multiple stages of muscle stem-cell biology and homeostasis. Satellite cells, the resident stem cells, execute the regenerative program (activation, proliferation, differentiation, fusion, self-renewal) (Figure 2) under intrinsic myogenic control and an extrinsic niche whose extracellular matrix and mechanical properties they read [45,46]. Fibro-adipogenic progenitors (FAPs) provide matricellular support, and their dysregulation drives the fibrosis and fatty infiltration of wasting [47]. Aging degrades this support, for example, by reducing FAP-derived WISP1 [48]. Piezo1 is increasingly recognized as a sensor that reads the mechanical component of this niche. Unlike the convergence model in Section 6, the relationships in this section are experimentally validated in skeletal muscle and have been consolidated in three recent reviews [49,50,51]. Table 1 summarizes the principal studies.

### 3.1. Satellite-Cell Quiescence and Senescence

Satellite cells use mechanical cues to balance quiescence and activation. Piezo1 mechano-signaling maintains quiescence and protects against senescence. In its absence, cells activate prematurely, proliferate and differentiate poorly, and fail to replenish the pool [19]. Mechanistically, Piezo1 loss upregulates T-type calcium channels, raising calcium entry and driving NOX4 via protein kinase C. The resulting ROS and DNA damage activate p53/p21 senescence, which p53 inhibition rescues [19]. Because mechano-signaling wanes with inactivity, this axis links disuse and aging to the accumulation of senescent cells and the fall in satellite-cell numbers seen in aged muscle [19]. Piezo1 also organizes quiescent satellite cells into responsive, intermediate, and sensory states, and shifts between them to regulate regeneration. Activation primes the responsive state, whereas deletion shifts cells toward the less responsive state seen in dystrophic muscle [20]. Together, one study defines the NOX4-ROS-p53 pathway that degrades the pool, and the other shows the same loss is reflected in satellite-cell morphology and readiness.

### 3.2. Regenerative Division and Mitotic Fidelity

Piezo1 also supports the mechanics of division, mediating the spontaneous, membrane-tension-driven calcium influx in satellite cells that is required for regeneration [21]. In satellite-cell-specific knockout mice, Piezo1 deletion delays regeneration after injury, partly through a mitotic defect with impaired chromosome segregation linked to disrupted Piezo1-Rho signaling [21]. Independently, a splicing-factor pathway centered on SRSF1 is likewise required for faithful spindle organization and prevention of premature senescence [52] so that faithful division depends on both mechanical (Piezo1-Rho) and post-transcriptional (splicing) control. Piezo1 thus governs both whether a satellite cell activates and the fidelity of its expansion, which sets the size and quality of the regenerative pool.

### 3.3. Myoblast Fusion and Terminal Differentiation

Piezo1 rises during differentiation and must be finely tuned. Its knockdown suppresses myoblast fusion and yields smaller myotubes with reduced fusogenic machinery, whereas activation raises stretch-evoked calcium and enhances fusion [22]. Consistent with this, Yoda1 stimulates differentiation and fusion of precursors within a defined time window while sparing mature myofibers and neuromuscular transmission, marking Piezo1 as a stage-specific, tractable lever on differentiation [23]. This fusion phenotype matters here because fusion is the functional output on which the Section 6 model is anchored.

This regulation is context-dependent, and the literature contains an instructive tension. Whereas Piezo1 knockdown suppresses fusion in normal myoblasts [22] and Yoda1 activation promotes differentiation in healthy precursors [23], the situation is reversed in Duchenne muscular dystrophy: Piezo1 is upregulated in patient and mdx muscle, and GsMTx4 inhibition curbs proliferation while promoting differentiation, increasing myofiber cross-sectional area and reducing centrally nucleated fibers [26]. These divergent outcomes are best reconciled by an optimal range for Piezo1 activity rather than simple maximization [22]. Both insufficient and excessive signaling impair progression from proliferation to differentiation, and the optimum differs between healthy regeneration and the calcium-overloaded dystrophic fiber.

### 3.4. Mechanical Loading, Unloading, and Atrophy

In adult muscle, Piezo1 couples mechanical state to mass. By calcium bioimaging, immobilization lowered cytosolic calcium below its basal level, accompanied by downregulation of Piezo1 [24]. Here, a decrease in calcium from baseline, rather than a rise, triggers the program, inverting the usual logic of calcium signaling [24]. Acute Piezo1 disruption induces a KLF15- and IL-6-dependent atrophy program. Neutralizing IL-6 protects against immobilization atrophy, and the Piezo1/KLF15/IL-6 axis was validated in human samples [24]. Reciprocally, muscle-specific Piezo1 overexpression prevents immobilization-induced loss of mass and cross-sectional area, and systemic overexpression was well tolerated, supporting the feasibility of Piezo1 activation in vivo [25]. Bone-derived signaling reinforces this protection. A Piezo1/osteocalcin/irisin axis in bone attenuates disuse atrophy through the muscle receptor Gprc6a and Fndc5/irisin, extending Piezo1’s anti-atrophic reach to inter-tissue endocrine control [53]. On this basis, Piezo1 has been proposed as a therapeutic target for disuse atrophy [3]. These studies cast Piezo1 as a bidirectional rheostat converting mechanical history into anabolic or catabolic output.

### 3.5. Loading-Induced Hypertrophy Versus Regeneration

A caveat about cellular context is warranted. Satellite-cell dynamics differ markedly between injury regeneration and load-induced hypertrophy: in regeneration, satellite cells run a stepwise program to rebuild myofibers, whereas in load-induced hypertrophy, their nuclei are added directly to existing fibers without the full sequence [54]. Most Piezo1-muscle evidence comes from regeneration and differentiation models, and its role in the accretion-based behavior of physiological hypertrophy is comparatively underexplored. This cautions against transferring conclusions from regeneration models directly to the exercise loading that is most relevant to sarcopenia.

Piezo1 also sits within a broader mechanosensory ensemble. Load-induced hypertrophy depends substantially on mTORC1: mechanical stimuli activate it, and deletion of its component raptor shows that it is necessary for hypertrophy but dispensable for the acute rise in protein synthesis [55,56]. Piezo1 is thus one node, alongside integrin adhesions and costameres, the dystrophin-glycoprotein complex, mTORC1, and other force-gated channels such as Piezo2 and TRP members, whose relative contributions are context-dependent [32,40]. The convergence model does not claim that Piezo1 is the sole mechanosensor; it asks whether the calcium-initiated Piezo1 arm intersects the noncoding RNA program, a question made tractable by Piezo1’s defined genetic and pharmacological handles. A useful way to separate their contributions is by the primary variable each sensor reports and the timescale on which it acts. Integrin-based adhesions and the costamere transmit lateral force between the sarcomere and the extracellular matrix and, through focal-adhesion kinase and downstream mTORC1, set the slow, hypertrophic protein-synthesis response to sustained loading. The dystrophin-glycoprotein complex stabilizes the sarcolemma against contraction-induced stress and, when disrupted, as in dystrophy, converts normal loading into damaging calcium entry. Against this background, Piezo1 is distinguished by its speed and its readout: it converts an acute change in membrane tension into an immediate calcium transient, placing it upstream of the calcium-dependent transcriptional and post-transcriptional events that are the focus of this review. Piezo2 and TRP-family channels overlap with Piezo1 in mechanosensitivity, but differ in inactivation kinetics and tissue distribution, and their specific roles in myogenesis remain comparatively undefined. In physiological loading, these systems act in concert rather than in isolation, and the extent to which the Piezo1 arm operates independently of, or in series with, integrin-mTORC1 signaling in vivo is not yet resolved.

## 4. From Piezo1 Calcium Signaling to the Myogenic Transcriptional Program

**Figure 3:**
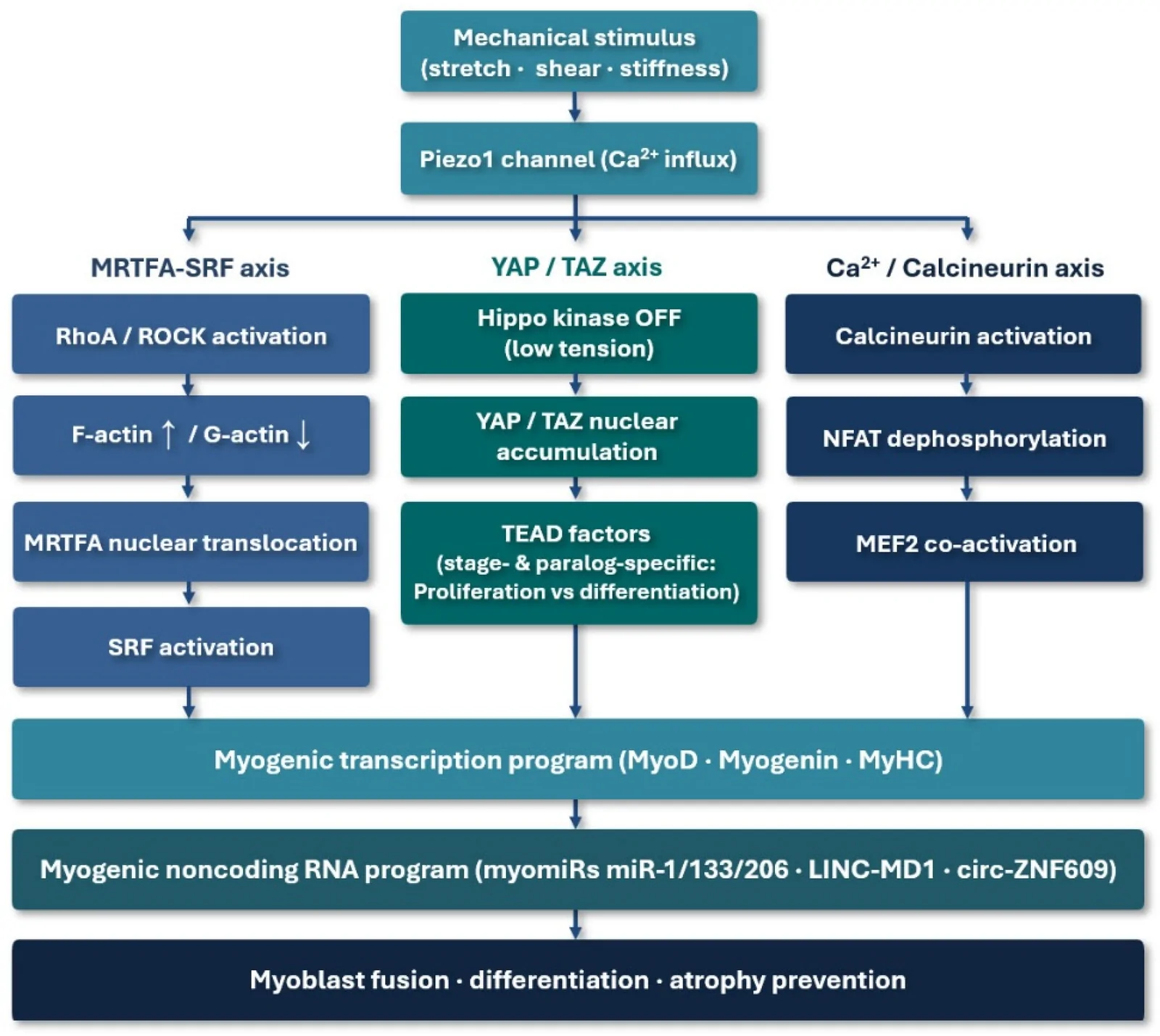
Piezo1 mechanotransduction signaling cascade in skeletal muscle. Piezo1-evoked Ca^2+^ influx feeds three transcriptional arms validated in muscle or adjacent tissue—RhoA/ROCK-actin-MRTFA-SRF, Hippo-YAP/TAZ-TEAD, and calcineurin/NFAT-MEF2—that converge on the myogenic transcription program. The YAP/TAZ arm is stage- and paralog-specific: YAP predominantly promotes satellite-cell proliferation and restrains differentiation, so its input to the myogenic program is context-dependent (Section 4.2).

How might a mechanical signal sensed at the membrane reach the myogenic gene program and the noncoding RNA program in the nucleus (Figure 3)? The route is provided by two interconnected, mechanically responsive transcriptional systems that act downstream of Piezo1-evoked calcium and cytoskeletal remodeling. Both are well characterized in muscle and converge on the core myogenic regulators.

### 4.1. The Actin-MRTFA-SRF Axis

Piezo1-mediated calcium influx engages RhoA/ROCK signaling and actin polymerization, as demonstrated for Piezo1-Rho signaling in satellite cells [21]. Actin dynamics are read out directly by myocardin-related transcription factor A (MRTFA, also MAL/MKL1): in resting cells, MRTFA is held in the cytoplasm through binding of monomeric G-actin to its N-terminal RPEL motifs, and when Rho-driven polymerization depletes the G-actin pool, MRTFA is released, accumulates in the nucleus, and partners with serum response factor (SRF) to activate cytoskeletal and contractile gene programs [57]. This actin-MRTFA-SRF module is a general mechanism for transducing cytoskeletal state into transcription, coupling the physical state of the actin cytoskeleton to a defined transcriptional output [58]. Genome-wide analyses have further shown that SRF, partitioned between MRTF and ternary-complex-factor (TCF) cofactors, selects distinct target-gene sets, with Rho-actin signaling to MRTF dominating the immediate transcriptional response to serum and mechanical stimulation [59]. In muscle, this module is the most direct relay between Piezo1- and SRF-dependent outputs, including, as developed below, the SRF-dependent myomiRs, whose transcription would be expected to track MRTFA-SRF activity and, therefore, the mechanical state sensed by Piezo1. The cytoskeletal arm of mechanotransduction is reinforced by integrin-based adhesion signaling. In C2C12 myoblasts, focal adhesion kinase (FAK) activation is required for the stretch-induced alignment and enhanced differentiation that accompany cyclic mechanical strain, linking adhesion-based force sensing to the myogenic program in parallel with Piezo1 [60]. That a Piezo1-calcium signal can directly reshape the actin cytoskeleton has been shown directly: in articular chondrocytes, elevated Piezo1 activity raises resting calcium and rarefies the F-actin network [39], providing cross-tissue proof of principle for the Piezo1-calcium-to-actin step on which the muscle model depends.

### 4.2. YAP/TAZ Mechanotransduction in Muscle

In parallel, the transcriptional coactivators YAP and TAZ act as mechanosensors that respond to cytoskeletal tension, extracellular matrix stiffness, and cell geometry, accumulating in the nucleus under high mechanical load to drive proliferation- and matrix-associated programs [61]. In skeletal muscle, their roles are stage- and paralog-specific. YAP is low in quiescent satellite cells and rises upon activation. Enforced YAP maintains Pax7+/MyoD+ cells, promotes proliferation while preventing differentiation, and acts through TEAD factors on MCAT promoter elements to do so [62]. TAZ shares the pro-proliferative function, but, unlike YAP, switches to a myogenic differentiation-enhancing role at later stages via TEAD4, so that the two Hippo effectors have overlapping yet divergent outputs [63]. Consistent with a net-restraining role for upstream Hippo kinases, suppression of the core component Salvador (SAV1) in muscle drives nuclear YAP and accelerates regeneration and angiogenesis in injured and ischemic muscle [64]. At the same time, Mst1/2 activity is required for YAP phosphorylation, which permits satellite-cell differentiation [65]. The integration with the core myogenic machinery is increasingly clear: VGLL4 acts as a coactivator of TEAD4 to promote myogenin transactivation and stabilize MyoD-TEAD4 interactions during differentiation [66], and the Hippo-YAP/TAZ pathway has been directly implicated in muscle aging and sarcopenia [67]. These studies place YAP/TAZ-TEAD at the convergence of mechanical input and the MyoD/myogenin program.

### 4.3. Calcium-Dependent Transcriptional Effectors

The actin- and Hippo-based routes are complemented by a more direct consequence of Piezo1 activation, namely the calcium signal itself. Sustained changes in intracellular calcium are decoded by the phosphatase calcineurin, which dephosphorylates NFAT transcription factors and, in combination with MEF2, selectively activates slow-fiber and other activity-dependent gene programs in skeletal muscle [68]. This calcineurin-NFAT-MEF2 pathway is the classical link between the calcium transients generated by motor-nerve activity and fiber-type-specific transcription [68,69], and because Piezo1 is a calcium-permeable channel, it is positioned to feed this pathway in parallel with the cytoskeletal route. MEF2, in turn, is a direct transcriptional driver of the myomiR loci (Section 5), providing a second, calcium-based path by which Piezo1 activity could, in principle, reach the noncoding RNA layer. Beyond calcineurin/NFAT, the MEK5–ERK5 arm of MAPK signaling is itself required for myogenic differentiation and myogenin induction [70]; whether Piezo1-evoked calcium feeds this arm in muscle, as it feeds calcineurin/NFAT, remains to be tested.

### 4.4. Convergence on Myogenic Outputs

Both the MRTFA-SRF and the YAP/TAZ modules ultimately converge on the muscle differentiation program (MyoD, myogenin, and myosin heavy chain) and on myoblast fusion. The YAP arm of this convergence has now received direct support in muscle. In dystrophic myoblasts, modulation of Piezo1 activity with GsMTx4 raises the YAP nuclear-to-cytoplasmic ratio in a manner that correlates with nuclear myogenin localization [26], providing an experimental link from Piezo1 activity through YAP to a core myogenic transcription factor. For this review, the key point is that the same SRF- and TEAD-dependent machinery also governs transcription of the principal myogenic noncoding RNAs (Section 5). The signaling architecture required to relay a Piezo1-derived mechanical signal to the ncRNA layer is therefore already present and, in part, experimentally substantiated in muscle. What has not been tested is whether Piezo1 activation actually modulates those ncRNAs (Section 6). The convergence argument therefore rests on two transcriptional bridges from Piezo1 to the myogenic program: SRF-dependent control of the myomiRs via the actin-MRTFA axis and MEF2- and calcineurin/NFAT-linked control of myogenic gene expression via the calcium arm.

## 5. Noncoding RNA Regulation of Myogenesis

### 5.1. A Layered Post-Transcriptional Control System

Skeletal myogenesis is not only governed by the canonical myogenic transcription factors (MyoD, Myf5, myogenin, MEF2), whose hierarchical action specifies and differentiates muscle from embryonic somite to adult satellite-cell [71,72,73], but also by an interlocking layer of noncoding RNAs that sharpen the timing, amplitude, and reversibility of the program. Because the core factors act within a chromatin environment shaped by Polycomb- and Trithorax-group complexes during satellite-cell activation [74], noncoding RNAs frequently act by guiding such chromatin modifiers or by titrating the factors and microRNAs that control these loci. Three classes are firmly established in muscle, microRNAs, long noncoding RNAs, and circular RNAs, each linked to myogenic differentiation, injury repair, and muscle disease [75]. Critically, the best-characterized members of each are transcriptionally tied to the same SRF/MEF2 machinery that lies immediately downstream of Piezo1-evoked mechano-signaling, making the myogenic ncRNA program a plausible target of mechanical input rather than a parallel, disconnected system.

### 5.2. Myogenic microRNAs (myomiRs)

The muscle-enriched miRNAs miR-1, miR-133, and miR-206 are the prototypical myomiRs. MicroRNAs are ~22-nucleotide regulators that silence target mRNAs by seed-pairing, predominantly within 3′ untranslated regions [76,77,78]. The myomiRs are transcribed from bicistronic clusters under the control of SRF, MyoD, and MEF2 and exert opposing, complementary effects on the myoblast decision to proliferate or differentiate [14]. miR-1 promotes differentiation, in part by targeting the transcriptional repressor HDAC4, whereas miR-133 favors proliferation, in part by repressing SRF itself, so that their shared, SRF-dependent transcription couples a single upstream signal to both arms of the proliferation-differentiation switch and embeds a feedback element within it [14]. Beyond this core trio, a broader myomiR family, including miR-208a/b and miR-499 encoded within myosin genes, together with miR-486, links contractile-gene expression to fiber-type identity and muscle performance, and an extensive catalog of myomiR target genes spanning every phase of myogenesis has now been compiled [15,79]. Because SRF activity is itself set by the actin-MRTFA axis that Piezo1 drives (Section 4), the myomiR cluster is the most direct candidate node through which a mechanical signal could be relayed to the post-transcriptional layer.

The disease relevance of these myomiRs is well established and directly motivates the therapeutic argument developed later. miR-206, the most muscle-restricted member, is strongly induced after denervation and nerve injury, where it is required for efficient regeneration of neuromuscular synapses. Genetic loss of miR-206 accelerates disease progression in a mouse model of amyotrophic lateral sclerosis, an effect mediated in part through histone deacetylase 4 and fibroblast growth factor signaling [80]. In dystrophic muscle, forced overexpression of miR-206 in mdx mice acts in a pleiotropic manner, simultaneously targeting multiple transcripts to favor satellite-cell differentiation, neuromuscular-junction maintenance, and utrophin upregulation, while dampening inflammation [81]. It is also overexpressed in the muscle of patients with myotonic dystrophy type 1 [82]. miR-486, likewise reduced in muscular dystrophy, can partially rescue dystrophic and cancer-associated muscle dysfunction when restored, in part by normalizing PI3K/AKT and inflammatory signaling [83]. These examples show that myomiRs are tractable effectors whose manipulation alters disease course, rather than passive readouts of the myogenic program. This is an important premise for the RNA-based strategies discussed in Section 7. This responsiveness also extends to neural input. Profiling of denervated muscle shows coordinated changes in miR-206 and other miRNAs that feed atrophy-related pathways (HDAC4-follistatin, IGF1/PI3K/Akt/FoxO-atrogin-1/MuRF1) and differ between fast and slow fibers, linking the myomiR network to the loss of motor-nerve activity that characterizes aging and disuse [84]. A concrete therapeutic corollary follows. Exosome-delivered miR-29, which represses YY1 and TGF-beta signaling, lowers the atrogenes MuRF1 and atrogin-1 and attenuates muscle wasting in mice [85], showing that delivering a single muscle-relevant miRNA can shift the atrophy program in vivo.

### 5.3. Long Noncoding RNAs

Long noncoding RNAs add a further layer, acting through chromatin modification, transcription-factor titration, and microRNA sponging [75,86,87]. The muscle-specific lncRNA LINC-MD1, the host transcript of miR-133b, acts as a competing endogenous RNA, sequestering miR-133 and miR-135 to de-repress the differentiation regulators MAML1 and MEF2C and thereby control the timing of the myogenic program [12]. A second mechanism is chromatin-based: the abundant lncRNA Malat1 restrains differentiation by recruiting the methyltransferase Suv39h1 to MyoD-binding loci to impose repressive H3K9 trimethylation, a brake that is released when the pro-myogenic miR-181a targets Malat1 for degradation, thereby licensing MyoD transactivation [88]. Similarly, Neat1 recruits the Polycomb component Ezh2 to myogenic promoters, promoting myoblast proliferation while suppressing differentiation [89], and the promoter-associated lncRNA Myoparr activates neighboring myogenin expression and myogenic-microRNA output to drive differentiation, while also promoting denervation-induced atrophy, illustrating that a single myogenic lncRNA can act in both differentiation and wasting [90]. For the mechanical theme of this review, at least one muscle lncRNA is directly mechanoresponsive: lncMUMA is the most strongly downregulated lncRNA during mechanical-unloading-induced atrophy, and its enforced expression, functioning as a miR-762 sponge to restore MyoD, both prevents and reverses established atrophy in hindlimb-suspended mice [91]. The lncMUMA transcript therefore provides an existence proof that mechanical state can be transduced into a myogenic lncRNA, and that such an lncRNA can be exploited therapeutically. It provides the closest current analog to the mechanically coupled noncoding node hypothesized here, albeit without a defined Piezo1 link.

### 5.4. Circular RNAs

Circular RNAs, generated by back-splicing and characterized by covalently closed structures and high stability, add a further dimension. Once dismissed as splicing errors, they are now recognized as an abundant, conserved, often tissue-specific class, some acting as potent microRNA sponges, most famously ciRS-7/CDR1as [92,93,94,95]. In muscle, circ-ZNF609, from the ZNF609 locus, controls myoblast proliferation and, unusually, contains a translatable open reading frame, blurring the line between noncoding scaffold and coding template [13]. A mechanistically complete example is circFgfr2, conserved across mammals, which sponges miR-133 to relieve repression of the Map3k20/JNK/MAPK pathway within a Klf4-dependent feedback loop, inhibiting proliferation while promoting differentiation and regeneration in C2C12 cells and mice [96]. It is instructive because it links a circRNA directly to a core myomiR (miR-133), a coupling that could be modulated by mechanical input. Because circRNA biogenesis is sensitive to splicing-factor activity and cellular state, sustained mechanical signaling could, in principle, reshape the circRNA repertoire during myoblast differentiation. Consistent with the importance of splicing control in satellite cells, the splicing factor SRSF1 is required to maintain satellite-cell proliferation and prevent premature senescence. Its loss leads to mitotic defects and cell cycle arrest [52]. Since back-splicing competes with canonical splicing, any input that alters splicing-factor activity could bias circRNA production alongside linear transcripts.

### 5.5. From a Parallel Program to a Mechanically Coupled One

The myogenic noncoding RNA program is both well defined and, through its SRF/MEF2 dependence, structurally positioned to receive input from Piezo1. What is missing is direct evidence that mechanical activation of Piezo1 modulates these specific nodes in myocytes, as formalized in Section 6. For clarity, the ncRNAs discussed here can be grouped by their priority within the convergence model. The primary candidates are the myomiR cluster (miR-1/133/206), LINC-MD1, and circ-ZNF609, each of which are central to the proliferation-to-differentiation switch. The myomiRs are directly SRF/MEF2-dependent, whereas for LINC-MD1 and circ-ZNF609, this transcriptional tie is inferred from their position rather than established (Section 6.4). These are the nodes that the predictions in Section 6 are designed to test. Secondary candidates, expected to respond to or modify the same axis but also to be less directly positioned, include miR-486, the mechanoresponsive lncRNA lncMUMA, Myoparr, Malat1, Neat1, and circFgfr2. The remaining examples provide broader contextual evidence that the myogenic noncoding layer is extensive and disease-relevant, with the broader muscle noncoding RNA landscape surveyed in several complementary reviews [97,98]. This prioritization, rather than the breadth of the catalog, defines the experimentally tractable core of the convergence model.

## 6. The Emerging Piezo1-Noncoding RNA Axis in Skeletal Muscle

### 6.1. Precedent from Other Mechanosensitive Lineages

Although a direct regulatory link between Piezo1 and noncoding RNAs has not yet been demonstrated in skeletal myocytes, the underlying circuitry is well documented in other mechanically loaded tissues and in both regulatory directions. By a direct Piezo1-noncoding RNA link, we mean a specific, molecularly defined relationship in which either Piezo1 activity sets the level or activity of a defined noncoding RNA through its immediate signaling output or a defined noncoding RNA sets Piezo1 abundance or activity, most concretely through a microRNA base-pairing with the PIEZO1 3′UTR; this is distinct from a diffuse, multi-step correlation. In one direction, Piezo1 acts upstream of a microRNA. In vascular smooth muscle cells exposed to simulated microgravity, mechanical unloading activates Piezo1 and increases miR-582-5p, which promotes a synthetic phenotype with collagen deposition and arterial stiffening through PTEN/PI3K/Akt signaling; inhibition of miR-582-5p attenuates these changes [16]. In the opposite direction, a microRNA acts upstream of Piezo1. In human cardiac fibroblasts, miR-214-3p directly represses Piezo1 expression and channel activity and concurrently remodels lysyl-oxidase and mitochondrial programs [17]. The precedents most relevant to skeletal muscle lie within the musculoskeletal system and striated muscle itself: a Piezo1-Mohawk (Mkx) pathway links mechanical loading to transcriptional output in tendon, alongside a defined role for miR-140 in cartilage homeostasis [99,100], and, in cardiomyocytes, Piezo1-mediated mechanotransduction drives hypertrophy by perturbing calcium homeostasis to activate calpain and calcineurin [101], engaging the same calcineurin/NFAT effector arm that operates in skeletal muscle (Section 4.3). The same bidirectional logic, spanning miRNAs, lncRNAs, and circRNAs, has been documented, though as weaker analogies for myogenesis, in hepatocellular carcinoma, breast cancer, and glioblastoma, in overloaded chondrocytes, and in broader surveys of extracellular matrix stiffness sensing across liver, breast, bone, and cardiovascular tissue [102,103,104,105,106]. Across these systems, both the Piezo1-to-ncRNA and the ncRNA-to-Piezo1 directions are experimentally tractable. What is conspicuously absent from this catalog is skeletal muscle. Both regulatory directions are thus supported by precedent in non-muscle tissues, yet neither has been demonstrated in myocytes and both remain hypothesized here.

### 6.2. The Skeletal-Muscle Gap

**Figure 4:**
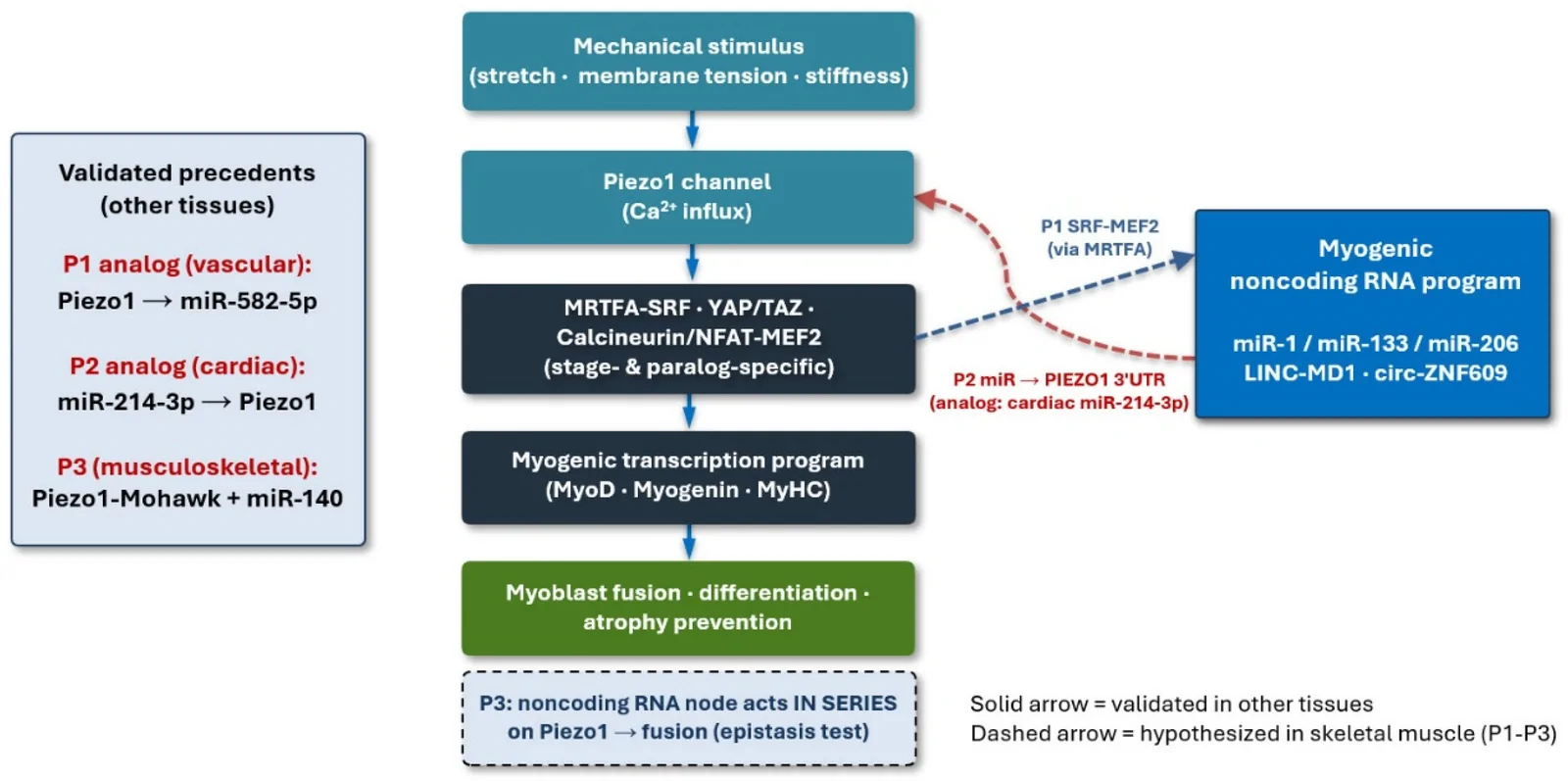
Proposed bidirectional Piezo1-noncoding RNA circuit in skeletal muscle. Solid arrows denote interactions validated in other tissues (vascular Piezo1-to-miR-582-5p; cardiac miR-214-3p-to-PIEZO1; musculoskeletal Piezo1-Mohawk and miR-140); dashed arrows denote the hypothesized skeletal-muscle links: P1 (Piezo1/SRF induction of myogenic noncoding RNAs), P2 (microRNA feedback onto the Piezo1 3′UTR), and P3 (the noncoding RNA node acting in series on fusion). The critical gap—no direct evidence in skeletal-muscle myocytes—and the C2C12/satellite-cell test platform are indicated, together with the top computed candidate miRNAs (Section 6.4, Table 3).

This wiring has not been tested in skeletal muscle, despite both halves of the circuit being independently well characterized there. On one side, Piezo1 governs satellite-cell quiescence and senescence [19], regenerative division [21], myoblast fusion [22], and disuse atrophy [24] (Table 1). On the other side, the myogenic noncoding RNA program, comprising the myomiRs [14,15], LINC-MD1, and circ-ZNF609 [13], is among the best-defined in any lineage. The convergence is mechanistically plausible rather than speculative because Piezo1-evoked calcium influx feeds the RhoA/ROCK-actin-MRTFA-SRF and YAP/TAZ axis that already drives myogenic transcription (Section 4). The machinery required to relay a mechanical signal from Piezo1 to noncoding RNA loci is therefore already present in muscle. What remains untested is whether the two layers are functionally coupled (Figure 4).

### 6.3. Testable Predictions

**Figure 5:**
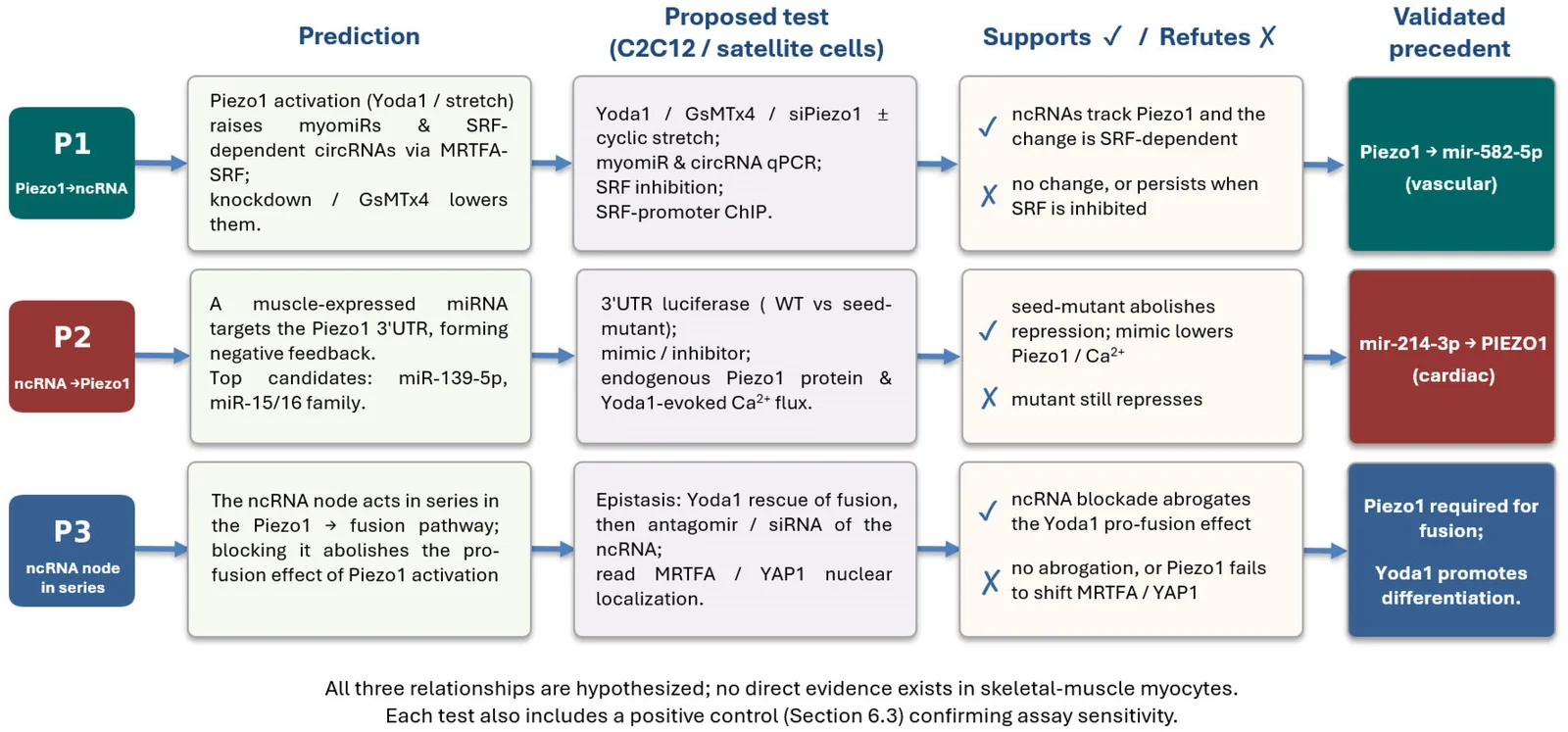
Experimental design for the three falsifiable predictions (P1–P3). For each prediction, the panel shows the hypothesized relationship, the proposed test in C2C12 or satellite cells, the criteria that would support or refute it, and the validated precedent from another lineage (Table 2). All three remain hypotheses, and each test includes a positive control confirming assay sensitivity (Section 6.3).

**Figure 6:**
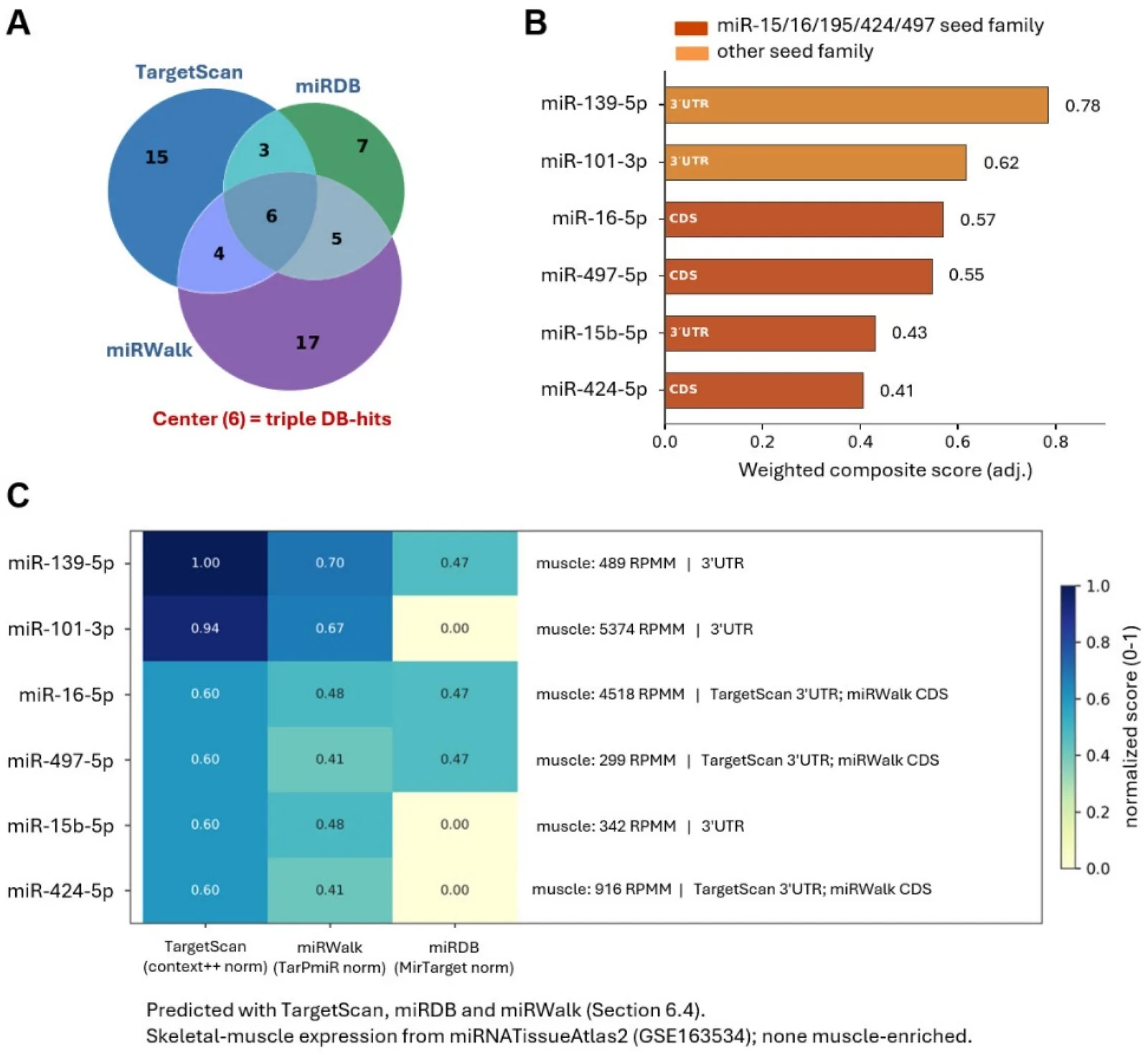
Unbiased three-tool prediction of PIEZO1 3′UTR-targeting microRNAs. (**A**) Overlap of microRNAs predicted to target the human PIEZO1 3′UTR by TargetScan, miRDB, and miRWalk; the six Triple-DB hits at the center are carried forward. (**B**) Weighted composite score of the six candidates, colored by seed family (the miR-15/16/195/424/497 family versus other families). The composite score is a display heuristic only; primary prioritization follows conserved-site count, context++ score, and skeletal-muscle expression (Section 6.4). (**C**) Per-tool normalized scores, with the best-site region and the skeletal-muscle expression of each candidate (RPMM, mean of four donor samples; miRNATissueAtlas2, GEO GSE163534). All six are expressed in skeletal muscle, yet none is muscle-enriched (Figure 7, Table 3). The shortlist is hypothesis-generating and requires the direct tests of Prediction 2 (Section 6.3).

**Figure 7:**
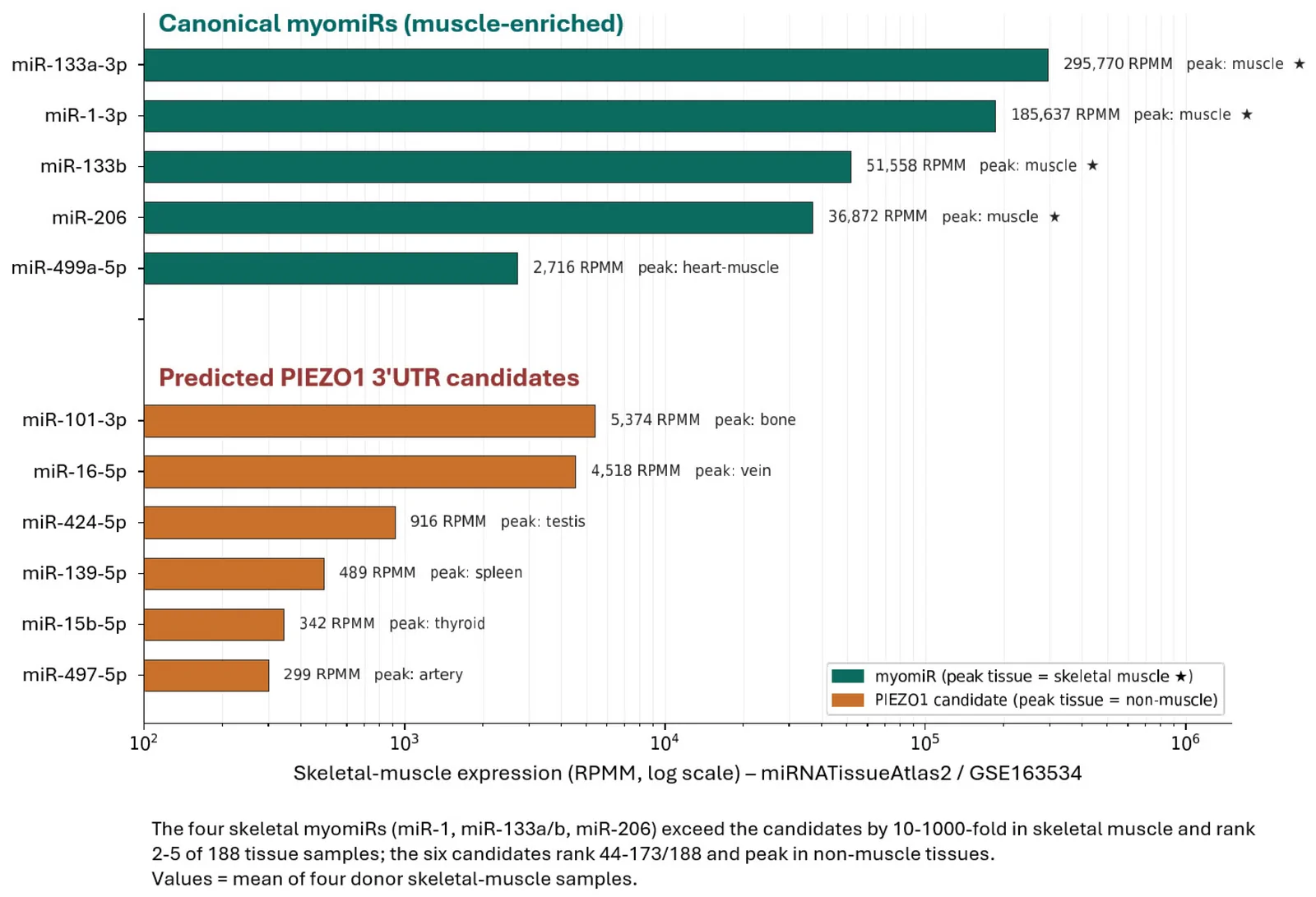
Predicted PIEZO1 3′UTR candidates are expressed in skeletal muscle but are not muscle-enriched, unlike canonical myomiRs. Skeletal-muscle expression (RPMM, mean of four donor samples; miRNATissueAtlas2, GEO GSE163534) for the six computed candidates and five canonical myomiRs, on a log scale. The skeletal myomiRs miR-1, miR-133a/b, and miR-206 exceed the candidates by 10–1000-fold and rank 2nd–5th of 188 tissue samples, with skeletal muscle as their peak tissue, whereas the candidates rank 44th–173rd and peak in non-muscle tissues (star = peak tissue is skeletal muscle). The abundance gap shows that the predicted candidates are minor, broadly expressed species in muscle, and must be confirmed by the direct tests of Prediction 2 (Table 3, Supplementary Table S2).

Three falsifiable predictions follow, each anchored to a validated precedent in another lineage and each addressable with established myogenic tools (Table 2, Figure 5). The circuit overview in Figure 4 and the per-prediction experimental design in Figure 5 together summarize the three predictions and their tests. All three remain hypotheses. No direct evidence currently exists in skeletal myocytes. Predictions 1 and 2 are logically independent, probing opposite directions of the circuit, whereas Prediction 3 is the functional consequence of Prediction 1 and is contingent on it. Predictions 1 and 3 rest on the firmest ground, the muscle-specific, SRF-driven myomiRs and the validated requirement for Piezo1 in fusion, whereas Prediction 2 is the most exploratory: the unbiased screen returns no muscle-specific Piezo1 targeter (Figure 6, Table 3), because the canonical myomiRs are not predicted to bind the Piezo1 3′UTR so that any feedback would involve a broadly expressed microRNA acting in the muscle context (Figure 7). This asymmetry sets the experimental priority: Predictions 1 and 3 are the primary tests of the framework, whereas Prediction 2 is presented as a secondary, more exploratory extension, with its computational candidate analysis presented in Figure 6 and Table 3.

**Prediction 1 (Piezo1 to ncRNA):** Piezo1 activation (Yoda1 or cyclic stretch) increases myomiR and SRF-dependent myogenic circRNA expression through MRTFA-SRF, while Piezo1 knockdown or GsMTx4 suppresses it. This mirrors the validated Piezo1-to-miR-582-5p relationship in vascular smooth muscle [16]. It can be tested in C2C12 cells (with Yoda1/GsMTx4/siPiezo1, with and without stretch), with SRF dependence established by pharmacological or genetic SRF inhibition and SRF promoter occupancy assessed by chromatin immunoprecipitation. The same design can probe the parallel calcium-MEF2 and ERK5/MAPK arms (Section 4.3), distinguishing which transcriptional route carries the signal. Because the myomiRs are co-transcribed from SRF-dependent loci yet act in opposing directions (miR-1 favoring differentiation, miR-133 proliferation), a shared rise in transcription need not produce a single functional output, and miR-133 repression of SRF could make the response self-limiting or biphasic, which time-resolved measurement should capture. The prediction fails if Piezo1 manipulation leaves these ncRNAs unchanged or if any change persists when SRF is inhibited.

**Prediction 2 (ncRNA to Piezo1):** A microRNA expressed in muscle directly targets the Piezo1 3′ untranslated region (UTR), forming a negative feedback loop that attenuates mechano-signaling, analogous to miR-214-3p repression of Piezo1 in cardiac fibroblasts [17]. Candidates should be selected by unbiased seed-match prediction rather than asserted a priori (Section 6.4), then tested with a Piezo1 3′UTR luciferase reporter (wild-type versus seed-mutant) and mimic/inhibitor, with confirmation at the level of endogenous Piezo1 protein and Yoda1-evoked calcium flux. The feedback is most consequential if the targeting microRNA is muscle-enriched or mechanoresponsive, so muscle expression should be weighted in candidate ranking (Section 6.4). Applied to the Piezo1 3′UTR, this pipeline nominates miR-139-5p, which carries the strongest predicted 3′UTR site (an 8mer), together with the broadly expressed miR-15/16/195/424/497 seed family, which shares a single 3′UTR site and includes the myogenic miR-424/322, as the leading candidates for these tests (Table 3). Because the unbiased screen returns no muscle-specific Piezo1 targeter, Prediction 2 resolves into two testable alternatives: either a muscle-enriched microRNA engages a non-canonical or lower-ranked Piezo1 site not captured by this pipeline, or one of these broadly expressed candidates provides the feedback within the muscle context. The luciferase and mimic tests distinguish these. The prediction fails if the seed-mutant reporter retains repression or if the mimic does not alter endogenous Piezo1 or calcium responses.

**Prediction 3 (functional serial node):** The Piezo1-ncRNA node operates in series within the F-actin-MRTFA-SRF/YAP1-MyoD/myogenin/MyHC pathway, such that interrupting the ncRNA node abolishes the pro-fusion effect of Piezo1 activation. This builds on the established requirement for Piezo1 in fusion [22] and the pro-differentiation effect of Yoda1 [23]. An epistasis design rescuing the fusion index with Yoda1 and then blocking the candidate ncRNA with an antagomir/siRNA would place the node upstream or downstream of MRTFA/YAP1 nuclear translocation. The prediction fails if ncRNA blockade does not abrogate the Yoda1 effect or if Piezo1 activation does not shift MRTFA/YAP1 localization in the first place.

### 6.4. A Note on Candidate Prioritization

**Methodological note:** Candidate microRNAs targeting the Piezo1 3′UTR were identified by an unbiased, intersection-based strategy rather than a priori. Human and mouse PIEZO1 3′UTR sequences were retrieved from RefSeq/Ensembl, and target sites were predicted independently with TargetScan, miRDB, and miRWalk, retaining only miRNAs returned by all three with mammalian seed conservation. This list was then cross-referenced with miRNAs reported to be expressed in skeletal myocytes (public myoblast/myotube small-RNA-seq and tissue-expression resources) to flag muscle-irrelevant candidates and rank them by site number, context++ score, and muscle expression. In the present analysis, the muscle-expression values are taken from the miRNATissueAtlas2 RPMM matrix (GEO GSE163534, mean of four donor skeletal-muscle samples; Table 3). This computed intersection, rather than any pre-selected list, populates the candidate set for Prediction 2, and Table 4 then serves as the positive-control set that a valid screen should recover. For lncRNAs and circRNAs, competing endogenous RNA potential was assessed with ENCORI/starBase and annotation was cross-referenced against circBase and circAtlas. The resulting shortlist is hypothesis-generating only. Each candidate requires direct experimental validation before any regulatory claim. The full list of predicted target sites, per-tool scores, seed conservation, and pipeline parameters for all 57 candidate microRNAs, together with the tool versions (TargetScan Release 8.0, miRDB MirTarget v4, miRWalk TarPmiR) and query date, is provided in Supplementary Table S1.

Applying this strategy to the human PIEZO1 3′UTR (NM_001142864) with TargetScan [111], miRDB [112], and miRWalk [113] returned six miRNAs predicted by all three tools (Triple-DB hits): miR-139-5p, miR-101-3p, miR-16-5p, miR-497-5p, miR-15b-5p, and miR-424-5p, all broadly conserved (Table 3, Figure 6). All six are expressed in skeletal muscle in the miRNATissueAtlas2 RPMM data (mean 299–5374 RPMM across four donor samples), but none is muscle-enriched, each peaking in a non-muscle tissue (Table 3). Four belong to the miR-15/16/195/424/497 seed family and share a single predicted 3′UTR site, so PIEZO1 may be co-targeted by several family members; among them, miR-424-5p (miR-322/424) has a defined myogenic role, making the family a particularly attractive Prediction-2 node. The strongest site lies in the 3′UTR for miR-139-5p, miR-101-3p, and miR-15b-5p (an 8mer for miR-139-5p and miR-101-3p), whereas for miR-16-5p, miR-497-5p, and miR-424-5p the strongest miRWalk site falls in the coding sequence, although all six retain a TargetScan-predicted 3′UTR seed match. By combined seed strength and cross-tool agreement, miR-139-5p is the leading single candidate (TargetScan −0.59 8mer, miRDB 82, miRWalk 3′UTR). Because several candidates are broadly expressed and target many transcripts, in particular the miR-15/16/195/424/497 family, their predicted interactions with the PIEZO1 3′UTR carry a higher risk of false positives. Consistent with this, in the miRNATissueAtlas2 RPMM data, all six are expressed in skeletal muscle (mean 299–5374 RPMM across four donors), yet none are muscle-enriched, each peaking in a non-muscle tissue (spleen, bone, vein, artery, thyroid, or testis), so a muscle-enriched or mechanoresponsive microRNA identified from a dedicated skeletal-muscle small-RNA-seq would be a preferable Prediction-2 candidate (Figure 7). Muscle-expression level, mechanoresponsiveness, and evolutionary conservation of the seed site should therefore weigh prioritization, and the direct luciferase and mimic tests of Prediction 2 are needed to distinguish genuine feedback from promiscuous targeting. These remain computational predictions that generate, but do not establish, the feedback of Prediction 2, and the muscle-expression filter should be confirmed against Tissue Atlas 2 or FANTOM5 before experimental prioritization.

**Experimental roadmap:** The predictions above acquire force only when paired with a concrete test design, expected effect direction and magnitude, controls, and an interpretation of negative results.

For Prediction 1, in differentiating C2C12 or primary human myoblasts, Yoda1 activation and Piezo1 knockdown should move miR-1/133/206, LINC-MD1, and circ-ZNF609 in opposite directions, with effect sizes comparable to direct manipulation of the MRTFA-SRF axis (commonly 1.5- to 3-fold for myomiRs). Essential controls include extracellular calcium chelation and ROCK or MRTFA inhibition to establish calcium- and actin-MRTFA dependence, together with parallel readouts of canonical SRF targets to confirm pathway engagement. Direct SRF or TEAD occupancy at the LINC-MD1 and circ-ZNF609 host loci has not been established, so the chromatin-immunoprecipitation step tests, rather than assuming, this link. A result showing that myomiRs remain unchanged despite confirmed Piezo1 activation, and SRF-target induction would falsify transcriptional coupling and redirect attention to post-transcriptional effects.

For Prediction 2, the candidates in Table 4 should be tested in myocytes by PIEZO1 3′UTR luciferase reporters with seed-mutation controls, by mimic and inhibitor titration against endogenous Piezo1 protein and Yoda1-evoked calcium influx, and by Argonaute pull-down to confirm physical engagement. A mimic is expected to lower Piezo1 protein and calcium response, while an inhibitor raises both. miR-214-3p is the highest-priority anchor because its direct repression of Piezo1 has already been validated in human cardiac fibroblasts, a stromal cell type of striated muscle tissue [17]. Failure to repress in myocytes, despite repression in the original system, would indicate context-dependent 3′UTR accessibility and would be informative in itself. More broadly, because none of the top-ranked candidates are muscle-enriched, the outcome sharpens into two explicit and mutually testable alternatives. Either a broadly expressed microRNA engages Piezo1 in the muscle context through context-dependent 3′UTR accessibility, in which case the regulator lies within the computed intersection but acts only where the muscle transcriptome permits, or the physiological regulator is a muscle-enriched microRNA that falls outside the top-ranked, seed-conserved set and would be recovered only by relaxing the intersection stringency or by direct screening in myocytes. Distinguishing these two possibilities is itself an informative experiment, and the breadth of expression of the leading candidates suggests that a Piezo1-targeting feedback loop may be a general feature of mechanosensitive tissues, with muscle specificity conferred by the surrounding myogenic transcription-factor and myomiR network rather than by a muscle-restricted microRNA.

For Prediction 3, time-resolved measurements should distinguish a feed-forward arrangement in which Piezo1 sets a myomiR that reinforces differentiation from a negative feedback arrangement in which a miRNA constrains Piezo1 to limit calcium overload. The disuse precedents for miR-337 in bone [110] and miR-582-5p in the vasculature [16] predict that an unloading stimulus should move at least one Piezo1-coupled miRNA in muscle. Testing this in a hindlimb-unloading or immobilization model is directly relevant to sarcopenia. All assays use standard myogenic models, commercial mimics, inhibitors, Yoda1, and established calcium imaging, so the program requires no new reagents and is limited to a tractable number of candidates.

### 6.5. Outlook

Establishing even one of these links would convert the Piezo1-noncoding RNA axis in skeletal muscle from analogy into evidence and would position mechanically tunable noncoding RNAs as candidate nodes for counteracting disuse atrophy and sarcopenia. The C2C12 system, in which the F-actin/G-actin balance, MRTFA-SRF and YAP1 activity, and myogenic output are all routinely measured, provides an immediate testbed for the predictions above. For primary satellite cells, in which introducing the large PIEZO1 cDNA has been difficult, baculovirus-mediated Piezo1 delivery now enables gain-of-function tests that complement the C2C12 experiments [114].

## 7. Disease and Therapeutic Implications

### 7.1. Sarcopenia and Disuse Atrophy

Sarcopenia is now primarily defined by low muscle strength, supported by low muscle quantity and quality, and is recognized as a generalized muscle disorder with major implications for falls, disability, and mortality [1]. Its pathophysiology is multifactorial, encompassing motor-unit remodeling, mitochondrial dysfunction, anabolic resistance, and impaired regeneration [2]. At the molecular level, muscle mass reflects a balance between anabolic and catabolic programs: the TGF-beta-family ligand myostatin acts as a negative regulator that limits muscle growth [115], while catabolic states converge on the FoxO-driven induction of the muscle-specific ubiquitin ligases atrogin-1/MAFbx and MuRF1, which target contractile proteins for proteasomal degradation [116,117]. Exercise opposes these catabolic pathways and remodels muscle through an integrated transcriptional and metabolic adaptation [118], and the mechanical loading it provides is the proximal stimulus that Piezo1 is positioned to sense. The Piezo1-centered mechanisms reviewed above map directly onto several of these axes. Reduced mechanical signaling, as occurs with physical inactivity and aging, drives satellite-cell senescence and pool depletion through the Piezo1-NOX4-p53 axis [19], while the Hippo-YAP/TAZ pathway downstream of mechanical input is itself implicated in muscle aging [67]. In the context of atrophy specifically, the Piezo1/KLF15/IL-6 program provides a defined causal pathway from immobilization to muscle wasting [24], and restoring Piezo1 activity prevents immobilization-induced atrophy [25]. A complementary bone-derived route, the Piezo1/osteocalcin/irisin axis, further protects muscle from disuse atrophy [53]. These convergent findings position Piezo1 as one well-defined and experimentally tractable node among the several mechanisms linking inactivity to age- and disuse-related muscle decline. They suggest that the loss of mechanical input in sedentary aging acts, at least in part, through Piezo1. By the same logic, the mechanical loading provided by resistance exercise, the most effective non-pharmacological countermeasure to sarcopenia, may exert part of its benefit by restoring Piezo1-dependent signaling. However, the satellite-cell behavior engaged by physiological hypertrophy differs from that of regeneration [54], and the precise contribution of Piezo1 to loading-induced hypertrophy remains to be defined. Any therapeutic inference for sarcopenia, therefore, rests on a mechanism validated in regeneration and disuse, but not yet in the exercise-induced loading it is meant to model, and this step requires direct confirmation. Exercise additionally counters two further features of the aging neuromuscular system that lie partly outside the satellite-cell compartment: it promotes reinnervation and preserves motor-unit organization in lifelong-active older adults, mitigating the denervation-driven fiber-type grouping characteristic of sarcopenic muscle [119], and it modulates the behavior of fibro-adipogenic progenitors, whose dysregulation drives the intramuscular fat infiltration and fibrosis that accompany muscle aging and disease [120]. A complete account of how mechanical input protects aging muscle will therefore need to integrate Piezo1-dependent satellite-cell signaling with these neural and stromal contributions.

### 7.2. Regenerative and Dystrophic Contexts

Piezo1 dysfunction is also relevant to impaired regeneration, and the dystrophic context illustrates both the promise and the complexity of targeting it. Satellite cells in dystrophic muscle adopt less responsive morphological states, and pharmacological reactivation of Piezo1 ameliorates these morphological and regenerative defects [20]. In Duchenne muscular dystrophy specifically, Piezo1 is upregulated in patients and mdx muscle, and inhibition with GsMTx4 promotes myogenic differentiation, increases myofiber cross-sectional area, reduces centrally nucleated fibers, and lowers pathological calcium load and CaMKII activation, identifying Piezo1 as a candidate therapeutic target in DMD [26]. Translating this into therapy, however, must confront the pharmacology of the tool used: GsMTx4 is a gating-modifier peptide with incomplete selectivity across mechanosensitive channels and limited oral bioavailability, so dose, local or muscle-targeted delivery, and off-target inhibition of Piezo1 in the vasculature and blood cells would all need to be resolved, and its benefit in dystrophic fibers must be weighed against the loss of the physiological, pro-differentiation Piezo1 signaling seen in healthy muscle [26]. This sits alongside the proposal, prompted by the Piezo1/KLF15/IL-6 work, that Piezo1 be regarded as a viable target for disuse atrophy [3]. The beneficial manipulation differs by context, Piezo1 inhibition in the calcium-overloaded dystrophic fiber [26] versus Piezo1 activation in disuse atrophy [25] and healthy differentiation [23], which shows that Piezo1 is best understood as a channel whose activity must be normalized to a context-specific optimum rather than uniformly enhanced or suppressed. The requirement for finely balanced Piezo1 activity, therefore, implies that the therapeutic window is narrow and direction-dependent and that the appropriate intervention will hinge on the underlying disease state.

### 7.3. Pharmacological and RNA-Based Strategies

This work suggests two therapeutic modalities. First is direct modulation of Piezo1. The agonist Yoda1 promotes differentiation and regeneration-associated responses [23,41], whereas the inhibitor GsMTx4 blunts pathological mechano-signaling and, combined with exercise, improves skeletal-muscle structure and motor function in a spinal-cord-injury model [42,121]. The beneficial direction of Piezo1 modulation is context-dependent, with activation favoring healthy regeneration and differentiation, but inhibition benefiting the calcium-overloaded dystrophic fiber (Section 7.2). Any such strategy must therefore be matched to the specific muscle state rather than applied as uniform activation or inhibition. These states are not a single continuum, and each has a distinct optimal Piezo1 activity. In acute injury-induced regeneration and in disuse or immobilization atrophy, where the problem is insufficient mechanical signaling, Piezo1 activation is the beneficial direction: it promotes satellite-cell differentiation and counters the loss of calcium-dependent anabolic signaling. In Duchenne muscular dystrophy, the logic is reversed: the fiber is already calcium-overloaded because of a fragile sarcolemma, and excess Piezo1 activity is harmful here, so partial inhibition or gating restraint is protective. Age-related sarcopenia is intermediate and heterogeneous, combining reduced loading, blunted satellite-cell responsiveness, and low-grade inflammation, so the optimal set-point is likely a restoration toward normal rather than maximal activation. Piezo1 is therefore best viewed not as uniformly beneficial or harmful, but as a channel whose activity must be normalized to a context-specific optimum, and any therapeutic strategy must be matched to the disease and cell state rather than applied as blanket activation or inhibition. Second is RNA-based intervention. If the predictions of Section 6 are borne out, mimics or inhibitors of the relevant myogenic noncoding RNAs could be used to bias the Piezo1-driven differentiation program, an approach that may permit muscle-selective tuning where direct channel modulation cannot. This is grounded in evidence rather than speculation because myogenic noncoding RNAs have already shown therapeutic activity in muscle disease models: restoring miR-206 or miR-486 improves dystrophic and cancer-associated muscle phenotypes [81,83], and enforced expression of the mechanoresponsive lncRNA lncMUMA reverses established unloading atrophy [91]. These precedents indicate that the noncoding layer is therapeutically addressable in muscle. Coupling it to a defined mechanosensor would enable engagement through mechanical or pharmacological control of Piezo1. Both strategies face the same two obstacles: the near-ubiquitous expression of Piezo1 raises the risk of off-target effects on the vasculature, blood cells, and other tissues, arguing for muscle-targeted or locally controlled delivery, and RNA therapeutics must overcome nuclease degradation, inefficient uptake, and off-target activity, for which advances in lipid nanoparticles, conjugates, and tissue-tropic carriers will be decisive. Several strategies could mitigate these limitations. First, tissue restriction of delivery, through intramuscular or limb-localized administration, muscle-homing peptide or antibody conjugates, and AAV serotypes with muscle tropism under muscle-specific promoters, could confine either channel modulators or RNA therapeutics to skeletal muscle and spare the vasculature and blood cells. Second, the RNA arm offers selectivity that direct channel drugs lack: because a myogenic microRNA or its target can be chosen for muscle-enriched expression, an oligonucleotide approach may achieve muscle specificity that a broadly expressed channel target cannot, provided that the delivery problem above is solved. Third, for the channel itself, biased or context-dependent modulators that act preferentially on the tension-gated state, rather than tonic openers, may reduce off-target effects, and gene-therapy approaches that adjust Piezo1 expression in a muscle-restricted manner offer a longer-term route to specificity. We present these as directions requiring validation rather than established solutions.

## 8. Limitations and Open Questions

Several limitations bound the synthesis presented here. First, the central proposition, a direct and bidirectional Piezo1-noncoding RNA axis in skeletal muscle, is, at present, an inference from adjacent systems rather than a fact. The specific molecular links in muscle remain to be demonstrated, and the predictions in Section 6 are offered precisely so that they can be tested and, if necessary, refuted. Second, much of the Piezo1-muscle literature relies on rodent models and a small number of cell systems. Species differences in Piezo1 regulation and pharmacology, as well as in myomiR biology, require human validation before therapeutic inferences are drawn. The near-absence of human data is the most concrete of these gaps. It is directly addressable: targeted studies in human muscle biopsies, including age-stratified cohorts and sarcopenic-versus-control comparisons, are needed to test whether the Piezo1-noncoding RNA relationships proposed here operate in human muscle [44]. Third, Piezo1 acts within a broader mechanosensory ensemble that includes Piezo2, TRP channels, integrin-based adhesions, and the dystrophin-glycoprotein complex [32], and the relative contribution of Piezo1 to muscle mechanotransduction in vivo remains unresolved. Fourth, the noncoding RNA field is itself prone to over-interpretation: predicted miRNA-target interactions frequently fail experimental validation, and ceRNA effects depend on stoichiometry that is rarely measured. For these reasons, this review separates validated relationships from hypotheses throughout and treats rigorous, unbiased candidate selection (Section 6.4) and explicit falsification criteria (Section 6.3) as essential safeguards against the accumulation of unsupported claims in this area.

## 9. Conclusions and Future Directions

The evidence reviewed here rests on two solid pillars and one open bridge. Piezo1 is now firmly established as a mechanotransducer governing satellite-cell fate, regeneration, fusion, and responses to loading and unloading in skeletal muscle (Table 1), and the myogenic noncoding RNA program is among the best-characterized regulatory networks in the field. The bridge between them, a direct and bidirectional Piezo1-noncoding RNA axis in muscle, remains hypothetical. However, it is grounded in validated precedents from vascular, cardiac, and tendon tissue, as well as a signaling architecture, the actin-MRTFA-SRF and YAP/TAZ axes, that is already present in myocytes.

The immediate priority is therefore experimental rather than conceptual. The three falsifiable predictions set out in Section 6 (Table 2), addressed with the unbiased candidate-selection strategy described there and the established C2C12 differentiation system, provide a concrete route to converting analogy into evidence. The computed candidate microRNAs are an illustrative entry point for Prediction 2, not a conclusion of this review. Tissue-expression data show that the strongest predicted targeters are broadly expressed rather than muscle-enriched, thereby refining the hypothesis and sharpening experimental priorities rather than weakening the framework. Establishing even a single validated link between Piezo1 and noncoding RNA in skeletal muscle would open a new, mechanically tunable layer of control over myogenesis, with direct implications for disuse atrophy and sarcopenia. Separating what is known from what is proposed has been a guiding principle of this review, as that distinction will allow for the field to reliably build on the convergence outlined here.

## Figures and Tables

**Table 1:** Validated functions of Piezo1 in skeletal muscle.

Process	Model/Approach	Key Finding	Ref.
Satellite-cell quiescence/senescence	Piezo1-cKO mice; pharmacology	Piezo1 maintains quiescence; loss drives NOX4/ROS and p53-dependent senescence and pool depletion	[19]
Stem-cell morphological states	Imaging; Yoda1; dystrophic muscle	Piezo1 sets responsive vs. sensory states; activation primes regeneration and rescues dystrophic defects.	[20]
Regenerative division	Satellite-cell Piezo1-cKO; injury	Piezo1-Rho signaling supports mitosis and chromosome segregation; loss delays regeneration.	[21]
Myoblast fusion/differentiation	C2C12/satellite cells; knockdown; stretch	Fine-tuned Piezo1 is required for fusion; activation raises stretch-evoked Ca^2+^ and enhances fusion.	[22]
Differentiation (chemical)	Yoda1; myogenic precursors	Time-window Yoda1 activation promotes differentiation and fusion; myofibers are unaffected.	[23]
Disuse atrophy	Immobilization; cKO; human samples	Piezo1 downregulation triggers KLF15/IL-6 atrophy; IL-6 blockade protects	[24]
Atrophy prevention	Muscle-specific/systemic overexpression	Piezo1 overexpression prevents immobilization atrophy; systemically tolerated	[25]
Dystrophic muscle (DMD)	mdx mice; patient samples; GsMTx4/Yoda1	PIEZO1 upregulated; GsMTx4 inhibition promotes differentiation, raises YAP/MyoG, and reduces fiber damage	[26]

cKO, conditional knockout; ROS, reactive oxygen species. All entries are experimentally validated in skeletal muscle.

**Table 2:** Three falsifiable predictions for the skeletal-muscle Piezo1-ncRNA axis.

Prediction	Validated Precedent (Other Lineage)	Proposed Test (C2C12/Satellite Cells)	Falsification
P1: Piezo1 to ncRNA	Piezo1 to miR-582-5p, vascular smooth muscle [16]	Yoda1/GsMTx4/siPiezo1 +/- stretch; myomiR and circRNA qPCR; SRF inhibition; SRF ChIP	No change in ncRNAs, or the change persists despite SRF inhibition.
P2: ncRNA to Piezo1	miR-214-3p represses Piezo1, cardiac fibroblasts [17]	Unbiased 3′UTR seed prediction (top: miR-139-5p, miR-15/16 family); luciferase wt vs. mutant; mimic/inhibitor; endogenous Piezo1 and Ca^2+^	Repression retained by seed-mutant reporter; no effect on endogenous Piezo1/Ca^2+^
P3: serial node	Piezo1 required for fusion [22]; Yoda1 promotes differentiation [23]	Epistasis: Yoda1 rescue of fusion, then antagomir/siRNA block; MRTFA/YAP1 localization	ncRNA blockade fails to abrogate Yoda1 effect; Piezo1 does not shift MRTFA/YAP1

All three relationships are hypothesized, and no direct evidence exists in skeletal myocytes. Each test also includes a positive control that confirms assay sensitivity: a validated SRF-dependent myomiR induction for P1, the established miR-214–3p-Piezo1 3′UTR interaction for P2, and a Piezo1-independent pro-fusion cue for P3. ChIP, chromatin immunoprecipitation; UTR, untranslated region.

**Table 3:** Computed PIEZO1 3′UTR miRNA candidates from the unbiased three-tool intersection. miRNAs predicted by TargetScan, miRDB, and miRWalk (Triple-DB hits) for the human PIEZO1 3′UTR (NM_001142864), all broadly conserved, are listed in descending weighted composite score (a display heuristic only; see below), with each additionally annotated for skeletal-muscle expression. TargetScan, cumulative weighted context++ score (more negative = stronger); miRDB, MirTarget score (≥80 = high confidence); miRWalk, TarPmiR probability with the strongest predicted binding region. Predictions are hypothesis-generating and require the direct tests of Prediction 2. The muscle-expression column reports skeletal-muscle expression from the miRNATissueAtlas2 RPMM matrix (GEO GSE163534; mean of four donor skeletal-muscle samples). The weighted composite score is a heuristic aggregate shown for display only; primary prioritization follows conserved-site count, context++ score, and muscle expression (Section 6.4).

miRNA	Seed Family	TargetScan Context++ (Site)	miRDB	miRWalk TarPmiR (Region)	Skeletal-Muscle Expression
miR-139-5p	miR-139-5p	−0.59 (8mer)	82	0.92 (3′UTR)	Expressed in muscle (~489 RPMM); not muscle-enriched
miR-101-3p	miR-101-3p	−0.56 (8mer)	81	0.85 (3′UTR)	Expressed in muscle (~5374 RPMM); not muscle-enriched
miR-16-5p	miR-15/16/195/424/497	−0.38 (7mer-m8 + 8mer)	72	0.92 (CDS)	Expressed in muscle (~4518 RPMM); not muscle-enriched
miR-497-5p	miR-15/16/195/424/497	−0.38 (7mer-m8 + 8mer)	69	0.92 (CDS)	Expressed in muscle (~299 RPMM); not muscle-enriched
miR-15b-5p	miR-15/16/195/424/497	−0.38 (7mer-m8 + 8mer)	72	0.85 (3′UTR)	Expressed in muscle (~342 RPMM); not muscle-enriched
miR-424-5p	miR-15/16/195/424/497	−0.38 (7mer-m8 + 8mer)	69	0.85 (CDS)	Expressed in muscle (~916 RPMM); not muscle-enriched

**Table 4:** Experimentally reported miRNA-PIEZO1 interactions across mechanosensitive tissues, compiled as a literature-anchored candidate set for testing in skeletal muscle.

miRNA	Reported Effect on PIEZO1	Original System	Skeletal-Muscle Relevance	Ref.
**miRNAs reported to repress PIEZO1 (candidates for Prediction 2, ncRNA to Piezo1)**				
**miR-214-3p**	Represses Piezo1; mimic lowers protein and channel activity	Human cardiac fibroblasts	Expressed in striated muscle; regulates myoblast proliferation and differentiation; highest-priority anchor	[17]
**miR-103a-3p**	Targets Piezo1 3′UTR (luciferase); lowers Piezo1 protein	Endothelium (acute myocardial infarction)	miR-103/107 family broadly expressed in muscle	[107]
**miR-107**	Targets PIEZO1; lowers expression	Brain (neuroinflammation)	Shares the miR-103a seed family, supporting seed conservation	[108]
**miR-34a-5p**	Represses Piezo1 (dual-luciferase and ceRNA rescue)	Bone-marrow stromal and endothelial cells (osteonecrosis)	Increases with senescence and aging in muscle	[109]
**miR-139-5p**	Represses PIEZO1 (ceRNA sponge and rescue)	Glioma	Expressed in muscle	[105]
**miRNAs reported downstream of PIEZO1 (candidates for Predictions 1 and 3, Piezo1 to ncRNA)**				
**miR-337-3p**	Induced through the Piezo1-Hippo-YAP axis	Disuse-induced bone loss (hindlimb unloading)	Direct unloading model; sarcopenia-relevant	[110]
**miR-582-5p**	Induced by stretch-activated Piezo1	Vascular smooth muscle (simulated microgravity and aging)	Unloading- and aging-relevant	[16]
**miR-155-5p**	Induced by Piezo1 under mechanical overload	Chondrocytes (osteoarthritis)	Mechanical-overload senescence	[106]
**miR-625-5p**	Engaged in a matrix-stiffness feedback loop with Piezo1	Hepatocellular carcinoma	Matrix-stiffness sensing	[103]

All listed interactions were established in non-skeletal-muscle systems, and none have yet been validated in skeletal myocytes, which is the gap that this section addresses. Only the upper block, the miRNAs that repress PIEZO1, provides direct candidates for Prediction 2. The lower block collects miRNAs induced downstream of PIEZO1 (Predictions 1 and 3) and should not be read as direct 3′UTR-targeting candidates. This compiled set anchors, but does not replace the unbiased seed-conserved intersection among TargetScan, miRDB, and miRWalk described above. The entries are the published interactions that such a screen should recover.

## Data Availability

No new data were created or analyzed in this study. Data sharing is not applicable to this article.

## Supplementary Materials

The following supporting information can be downloaded at https://www.mdpi.com/article/10.3390/ijms27167084/s1.

## References

[1] Cruz-Jentoft A.J., Bahat G., Bauer J., Boirie Y., Bruyere O., Cederholm T., Cooper C., Landi F., Rolland Y., Sayer A.A. (2019). Sarcopenia: Revised European consensus on definition and diagnosis. Age Ageing.

[2] Larsson L., Degens H., Li M., Salviati L., Lee Y.I., Thompson W., Kirkland J.L., Sandri M. (2019). Sarcopenia: Aging-Related Loss of Muscle Mass and Function. Physiol. Rev..

[3] Jagasia R., Wagner K.R. (2022). Piezo1: Opening the way to preventing muscle atrophy. J. Clin. Investig..

[4] Coste B., Mathur J., Schmidt M., Earley T.J., Ranade S., Petrus M.J., Dubin A.E., Patapoutian A. (2010). Piezo1 and Piezo2 are essential components of distinct mechanically activated cation channels. Science.

[5] Coste B., Xiao B., Santos J.S., Syeda R., Grandl J., Spencer K.S., Kim S.E., Schmidt M., Mathur J., Dubin A.E. (2012). Piezo proteins are pore-forming subunits of mechanically activated channels. Nature.

[6] Ge J., Li W., Zhao Q., Li N., Chen M., Zhi P., Li R., Gao N., Xiao B., Yang M. (2015). Architecture of the mammalian mechanosensitive Piezo1 channel. Nature.

[7] Saotome K., Murthy S.E., Kefauver J.M., Whitwam T., Patapoutian A., Ward A.B. (2018). Structure of the mechanically activated ion channel Piezo1. Nature.

[8] Zhao Q., Zhou H., Chi S., Wang Y., Wang J., Geng J., Wu K., Liu W., Zhang T., Dong M.Q. (2018). Structure and mechanogating mechanism of the Piezo1 channel. Nature.

[9] Wang L., Zhou H., Zhang M., Liu W., Deng T., Zhao Q., Li Y., Lei J., Li X., Xiao B. (2019). Structure and mechanogating of the mammalian tactile channel PIEZO2. Nature.

[10] Li J., Hou B., Tumova S., Muraki K., Bruns A., Ludlow M.J., Sedo A., Hyman A.J., McKeown L., Young R.S. (2014). Piezo1 integration of vascular architecture with physiological force. Nature.

[11] Ranade S.S., Qiu Z., Woo S.H., Hur S.S., Murthy S.E., Cahalan S.M., Xu J., Mathur J., Bandell M., Coste B. (2014). Piezo1, a mechanically activated ion channel, is required for vascular development in mice. Proc. Natl. Acad. Sci. USA.

[12] Cesana M., Cacchiarelli D., Legnini I., Santini T., Sthandier O., Chinappi M., Tramontano A., Bozzoni I. (2011). A long noncoding RNA controls muscle differentiation by functioning as a competing endogenous RNA. Cell.

[13] Legnini I., Di Timoteo G., Rossi F., Morlando M., Briganti F., Sthandier O., Fatica A., Santini T., Andronache A., Wade M. (2017). Circ-ZNF609 Is a Circular RNA that Can Be Translated and Functions in Myogenesis. Mol. Cell.

[14] Chen J.F., Mandel E.M., Thomson J.M., Wu Q., Callis T.E., Hammond S.M., Conlon F.L., Wang D.Z. (2006). The role of microRNA-1 and microRNA-133 in skeletal muscle proliferation and differentiation. Nat. Genet..

[15] van Rooij E., Quiat D., Johnson B.A., Sutherland L.B., Qi X., Richardson J.A., Kelm R.J., Olson E.N. (2009). A family of microRNAs encoded by myosin genes governs myosin expression and muscle performance. Dev. Cell.

[16] Zhang J., Wang X., Fu Z., Xing C., Wang Z., Yang H., Li J., Liu M., Dong L., Zhang X. (2024). Long-term simulated microgravity fosters carotid aging-like changes via Piezo1. Cardiovasc. Res..

[17] Trevelyan C.J., MacCannell A.D.V., Stewart L., Tarousa T., Taylor H.A., Murray M., Bageghni S.A., Hemmings K.E., Drinkhill M.J., Roberts L.D. (2024). MiR-214-3p regulates Piezo1, lysyl oxidases and mitochondrial function in human cardiac fibroblasts. Matrix Biol..

[18] Asahara H. (2025). Molecular basis of individual locomotor function: Integrated understanding of gene expression regulation in the development and homeostasis of the musculoskeletal system. Proc. Jpn. Acad. Ser. B Phys. Biol. Sci..

[19] Peng Y., Du J., Gunther S., Guo X., Wang S., Schneider A., Zhu L., Braun T. (2022). Mechano-signaling via Piezo1 prevents activation and p53-mediated senescence of muscle stem cells. Redox Biol..

[20] Ma N., Chen D., Lee J.H., Kuri P., Hernandez E.B., Kocan J., Mahmood H., Tichy E.D., Rompolas P., Mourkioti F. (2022). Piezo1 regulates the regenerative capacity of skeletal muscles via orchestration of stem cell morphological states. Sci. Adv..

[21] Hirano K., Tsuchiya M., Shiomi A., Takabayashi S., Suzuki M., Ishikawa Y., Kawano Y., Takabayashi Y., Nishikawa K., Nagao K. (2023). The mechanosensitive ion channel PIEZO1 promotes satellite cell function in muscle regeneration. Life Sci. Alliance.

[22] Ortuste Quiroga H.P., Ganassi M., Yokoyama S., Nakamura K., Yamashita T., Raimbach D., Hagiwara A., Harrington O., Breach-Teji J., Asakura A. (2022). Fine-Tuning of Piezo1 Expression and Activity Ensures Efficient Myoblast Fusion during Skeletal Myogenesis. Cells.

[23] Bosutti A., Giniatullin A., Odnoshivkina Y., Giudice L., Malm T., Sciancalepore M., Giniatullin R., D’Andrea P., Lorenzon P., Bernareggi A. (2021). “Time window” effect of Yoda1-evoked Piezo1 channel activity during mouse skeletal muscle differentiation. Acta Physiol..

[24] Hirata Y., Nomura K., Kato D., Tachibana Y., Niikura T., Uchiyama K., Hosooka T., Fukui T., Oe K., Kuroda R. (2022). A Piezo1/KLF15/IL-6 axis mediates immobilization-induced muscle atrophy. J. Clin. Investig..

[25] Inoue T., Nishigaki T., Hirata Y., Nomura K., Sugawara K., Ogawa W. (2025). Effects of Systemic and Skeletal Muscle-Specific Overexpression of Piezo1. Kobe J. Med. Sci..

[26] Wang W., Huang M., Huang X., Ma K., Luo M., Yang N. (2025). GsMTx4-blocked PIEZO1 channel promotes myogenic differentiation and alleviates myofiber damage in Duchenne muscular dystrophy. Skelet. Muscle.

[27] Jiang Y., Yang X., Jiang J., Xiao B. (2021). Structural Designs and Mechanogating Mechanisms of the Mechanosensitive Piezo Channels. Trends Biochem. Sci..

[28] Zhao Q., Zhou H., Li X., Xiao B. (2019). The mechanosensitive Piezo1 channel: A three-bladed propeller-like structure and a lever-like mechanogating mechanism. FEBS J..

[29] Wang Y., Xiao B. (2018). The mechanosensitive Piezo1 channel: Structural features and molecular bases underlying its ion permeation and mechanotransduction. J. Physiol..

[30] Cox C.D., Bae C., Ziegler L., Hartley S., Nikolova-Krstevski V., Rohde P.R., Ng C.A., Sachs F., Gottlieb P.A., Martinac B. (2016). Removal of the mechanoprotective influence of the cytoskeleton reveals PIEZO1 is gated by bilayer tension. Nat. Commun..

[31] Ridone P., Vassalli M., Martinac B. (2019). Piezo1 mechanosensitive channels: What are they and why are they important. Biophys. Rev..

[32] Douguet D., Honore E. (2019). Mammalian Mechanoelectrical Transduction: Structure and Function of Force-Gated Ion Channels. Cell.

[33] Hyman A.J., Tumova S., Beech D.J. (2017). Piezo1 Channels in Vascular Development and the Sensing of Shear Stress. Curr. Top. Membr..

[34] Lim X.R., Harraz O.F. (2024). Mechanosensing by Vascular Endothelium. Annu. Rev. Physiol..

[35] Jia B.Z., Tang X., Rossmann M.P., Zon L.I., Engert F., Cohen A.E. (2025). Swimming motions evoke Piezo1-dependent Ca^2+^ events in vascular endothelial cells of larval zebrafish. Curr. Biol..

[36] Zarychanski R., Schulz V.P., Houston B.L., Maksimova Y., Houston D.S., Smith B., Rinehart J., Gallagher P.G. (2012). Mutations in the mechanotransduction protein PIEZO1 are associated with hereditary xerocytosis. Blood.

[37] Cahalan S.M., Lukacs V., Ranade S.S., Chien S., Bandell M., Patapoutian A. (2015). Piezo1 links mechanical forces to red blood cell volume. eLife.

[38] Li X., Han L., Nookaew I., Mannen E., Silva M.J., Almeida M., Xiong J. (2019). Stimulation of Piezo1 by mechanical signals promotes bone anabolism. eLife.

[39] Lee W., Nims R.J., Savadipour A., Zhang Q., Leddy H.A., Liu F., McNulty A.L., Chen Y., Guilak F., Liedtke W.B. (2021). Inflammatory signaling sensitizes Piezo1 mechanotransduction in articular chondrocytes as a pathogenic feed-forward mechanism in osteoarthritis. Proc. Natl. Acad. Sci. USA.

[40] Gao W., Hasan H., Anderson D.E., Lee W. (2022). The Role of Mechanically-Activated Ion Channels Piezo1, Piezo2, and TRPV4 in Chondrocyte Mechanotransduction and Mechano-Therapeutics for Osteoarthritis. Front. Cell Dev. Biol..

[41] Syeda R., Xu J., Dubin A.E., Coste B., Mathur J., Huynh T., Matzen J., Lao J., Tully D.C., Engels I.H. (2015). Chemical activation of the mechanotransduction channel Piezo1. eLife.

[42] Bae C., Sachs F., Gottlieb P.A. (2011). The mechanosensitive ion channel Piezo1 is inhibited by the peptide GsMTx4. Biochemistry.

[43] Evans E.L., Cuthbertson K., Endesh N., Rode B., Blythe N.M., Hyman A.J., Hall S.J., Gaunt H.J., Ludlow M.J., Foster R. (2018). Yoda1 analogue (Dooku1) which antagonizes Yoda1-evoked activation of Piezo1 and aortic relaxation. Br. J. Pharmacol..

[44] Miron T.R., Flood E.D., Tykocki N.R., Thompson J.M., Watts S.W. (2022). Identification of Piezo1 channels in perivascular adipose tissue (PVAT) and their potential role in vascular function. Pharmacol. Res..

[45] Yin H., Price F., Rudnicki M.A. (2013). Satellite cells and the muscle stem cell niche. Physiol. Rev..

[46] Han W.M., Jang Y.C., Garcia A.J. (2017). Engineered matrices for skeletal muscle satellite cell engraftment and function. Matrix Biol..

[47] Giuliani G., Rosina M., Reggio A. (2022). Signaling pathways regulating the fate of fibro/adipogenic progenitors (FAPs) in skeletal muscle regeneration and disease. FEBS J..

[48] Lukjanenko L., Karaz S., Stuelsatz P., Gurriaran-Rodriguez U., Michaud J., Dammone G., Sizzano F., Mashinchian O., Ancel S., Migliavacca E. (2019). Aging Disrupts Muscle Stem Cell Function by Impairing Matricellular WISP1 Secretion from Fibro-Adipogenic Progenitors. Cell Stem Cell.

[49] Mirzoev T.M. (2023). The emerging role of Piezo1 channels in skeletal muscle physiology. Biophys. Rev..

[50] Bernareggi A., Bosutti A., Massaria G., Giniatullin R., Malm T., Sciancalepore M., Lorenzon P. (2022). The State of the Art of Piezo1 Channels in Skeletal Muscle Regeneration. Int. J. Mol. Sci..

[51] Dienes B., Bazso T., Szabo L., Csernoch L. (2023). The Role of the Piezo1 Mechanosensitive Channel in the Musculoskeletal System. Int. J. Mol. Sci..

[52] Wang Z., Peng Q., Zhang Z., You X., Duan H., Sha R., Yuan N., Li Z., Xie Z., Han J. (2024). SRSF1 Is Crucial for Maintaining Satellite Cell Homeostasis During Skeletal Muscle Growth and Regeneration. J. Cachexia Sarcopenia Muscle.

[53] Wang Z., Jiang X., Sun X., Chen H., Jin J., Shi X. (2026). Mechanosensitive Piezo1/Osteocalcin/Irisin Axis Protects Against Disuse-Induced Muscle Atrophy. Adv. Sci..

[54] Fukada S.I., Higashimoto T., Kaneshige A. (2022). Differences in muscle satellite cell dynamics during muscle hypertrophy and regeneration. Skelet. Muscle.

[55] Goodman C.A. (2014). The role of mTORC1 in regulating protein synthesis and skeletal muscle mass in response to various mechanical stimuli. Rev. Physiol. Biochem. Pharmacol..

[56] You J.S., McNally R.M., Jacobs B.L., Privett R.E., Gundermann D.M., Lin K.H., Steinert N.D., Goodman C.A., Hornberger T.A. (2019). The role of raptor in the mechanical load-induced regulation of mTOR signaling, protein synthesis, and skeletal muscle hypertrophy. FASEB J..

[57] Miralles F., Posern G., Zaromytidou A.I., Treisman R. (2003). Actin dynamics control SRF activity by regulation of its coactivator MAL. Cell.

[58] Olson E.N., Nordheim A. (2010). Linking actin dynamics and gene transcription to drive cellular motile functions. Nat. Rev. Mol. Cell Biol..

[59] Esnault C., Stewart A., Gualdrini F., East P., Horswell S., Matthews N., Treisman R. (2014). Rho-actin signaling to the MRTF coactivators dominates the immediate transcriptional response to serum in fibroblasts. Genes. Dev..

[60] Andersen J.I., Pennisi C.P., Fink T., Zachar V. (2018). Focal Adhesion Kinase Activation Is Necessary for Stretch-Induced Alignment and Enhanced Differentiation of Myogenic Precursor Cells. Tissue Eng. Part A.

[61] Dupont S., Morsut L., Aragona M., Enzo E., Giulitti S., Cordenonsi M., Zanconato F., Le Digabel J., Forcato M., Bicciato S. (2011). Role of YAP/TAZ in mechanotransduction. Nature.

[62] Judson R.N., Tremblay A.M., Knopp P., White R.B., Urcia R., De Bari C., Zammit P.S., Camargo F.D., Wackerhage H. (2012). The Hippo pathway member Yap plays a key role in influencing fate decisions in muscle satellite cells. J. Cell Sci..

[63] Sun C., De Mello V., Mohamed A., Ortuste Quiroga H.P., Garcia-Munoz A., Al Bloshi A., Tremblay A.M., von Kriegsheim A., Collie-Duguid E., Vargesson N. (2017). Common and Distinctive Functions of the Hippo Effectors Taz and Yap in Skeletal Muscle Stem Cell Function. Stem Cells.

[64] Liu Q., Pan S., Liu S., Zhang S., Willerson J.T., Martin J.F., Dixon R.A.F. (2021). Suppressing Hippo signaling in the stem cell niche promotes skeletal muscle regeneration. Stem Cells.

[65] Yang J., Wang K., An Y., Wu R., Li J., Wang H., Dong Y. (2022). Mst1/2 Is Necessary for Satellite Cell Differentiation to Promote Muscle Regeneration. Stem Cells.

[66] Feng X., Wang Z., Wang F., Lu T., Xu J., Ma X., Li J., He L., Zhang W., Li S. (2019). Dual function of VGLL4 in muscle regeneration. EMBO J..

[67] Setiawan I., Sanjaya A., Lesmana R., Yen P.M., Goenawan H. (2021). Hippo pathway effectors YAP and TAZ and their association with skeletal muscle ageing. J. Physiol. Biochem..

[68] Chin E.R., Olson E.N., Richardson J.A., Yang Q., Humphries C., Shelton J.M., Wu H., Zhu W., Bassel-Duby R., Williams R.S. (1998). A calcineurin-dependent transcriptional pathway controls skeletal muscle fiber type. Genes. Dev..

[69] Schiaffino S., Reggiani C. (2011). Fiber types in mammalian skeletal muscles. Physiol. Rev..

[70] Dinev D., Jordan B.W.M., Neufeld B., Lee J.D., Lindemann D., Rapp U.R., Ludwig S. (2001). Extracellular signal regulated kinase 5 (ERK5) is required for the differentiation of muscle cells. EMBO Rep..

[71] Buckingham M., Bajard L., Chang T., Daubas P., Hadchouel J., Meilhac S., Montarras D., Rocancourt D., Relaix F. (2003). The formation of skeletal muscle: From somite to limb. J. Anat..

[72] Braun T., Gautel M. (2011). Transcriptional mechanisms regulating skeletal muscle differentiation, growth and homeostasis. Nat. Rev. Mol. Cell Biol..

[73] Bentzinger C.F., Wang Y.X., Rudnicki M.A. (2012). Building muscle: Molecular regulation of myogenesis. Cold Spring Harb. Perspect. Biol..

[74] Dilworth F.J., Blais A. (2011). Epigenetic regulation of satellite cell activation during muscle regeneration. Stem Cell Res. Ther..

[75] Zhao Y., Chen M., Lian D., Li Y., Li Y., Wang J., Deng S., Yu K., Lian Z. (2019). Non-Coding RNA Regulates the Myogenesis of Skeletal Muscle Satellite Cells, Injury Repair and Diseases. Cells.

[76] Lee R.C., Feinbaum R.L., Ambros V. (1993). The *C. elegans* heterochronic gene lin-4 encodes small RNAs with antisense complementarity to lin-14. Cell.

[77] Bartel D.P. (2009). MicroRNAs: Target recognition and regulatory functions. Cell.

[78] Ha M., Kim V.N. (2014). Regulation of microRNA biogenesis. Nat. Rev. Mol. Cell Biol..

[79] Horak M., Novak J., Bienertova-Vasku J. (2016). Muscle-specific microRNAs in skeletal muscle development. Dev. Biol..

[80] Williams A.H., Valdez G., Moresi V., Qi X., McAnally J., Elliott J.L., Bassel-Duby R., Sanes J.R., Olson E.N. (2009). MicroRNA-206 delays ALS progression and promotes regeneration of neuromuscular synapses in mice. Science.

[81] Amirouche A., Jahnke V.E., Lunde J.A., Koulmann N., Freyssenet D.G., Jasmin B.J. (2017). Muscle-specific microRNA-206 targets multiple components in dystrophic skeletal muscle representing beneficial adaptations. Am. J. Physiol. Cell Physiol..

[82] Gambardella S., Rinaldi F., Lepore S.M., Viola A., Loro E., Angelini C., Vergani L., Novelli G., Botta A. (2010). Overexpression of microRNA-206 in the skeletal muscle from myotonic dystrophy type 1 patients. J. Transl. Med..

[83] Wang R., Kumar B., Doud E.H., Mosley A.L., Alexander M.S., Kunkel L.M., Nakshatri H. (2022). Skeletal muscle-specific overexpression of miR-486 limits mammary tumor-induced skeletal muscle functional limitations. Mol. Ther. Nucleic Acids.

[84] Li G., Li Q.S., Li W.B., Wei J., Chang W.K., Chen Z., Qiao H.Y., Jia Y.W., Tian J.H., Liang B.S. (2016). miRNA targeted signaling pathway in the early stage of denervated fast and slow muscle atrophy. Neural Regen. Res..

[85] Wang H., Wang B., Zhang A., Hassounah F., Seow Y., Wood M., Ma F., Klein J.D., Price S.R., Wang X.H. (2019). Exosome-Mediated miR-29 Transfer Reduces Muscle Atrophy and Kidney Fibrosis in Mice. Mol. Ther..

[86] Ulitsky I., Bartel D.P. (2013). lincRNAs: Genomics, evolution, and mechanisms. Cell.

[87] Statello L., Guo C.J., Chen L.L., Huarte M. (2021). Gene regulation by long non-coding RNAs and its biological functions. Nat. Rev. Mol. Cell Biol..

[88] Chen X., He L., Zhao Y., Li Y., Zhang S., Sun K., So K., Chen F., Zhou L., Lu L. (2017). Malat1 regulates myogenic differentiation and muscle regeneration through modulating MyoD transcriptional activity. Cell Discov..

[89] Wang S., Zuo H., Jin J., Lv W., Xu Z., Fan Y., Zhang J., Zuo B. (2019). Long noncoding RNA Neat1 modulates myogenesis by recruiting Ezh2. Cell Death Dis..

[90] Hitachi K., Nakatani M., Takasaki A., Ouchi Y., Uezumi A., Ageta H., Inagaki H., Kurahashi H., Tsuchida K. (2019). Myogenin promoter-associated lncRNA Myoparr is essential for myogenic differentiation. EMBO Rep..

[91] Zhang Z.K., Li J., Guan D., Liang C., Zhuo Z., Liu J., Lu A., Zhang G., Zhang B.T. (2018). Long Noncoding RNA lncMUMA Reverses Established Skeletal Muscle Atrophy following Mechanical Unloading. Mol. Ther..

[92] Salzman J., Gawad C., Wang P.L., Lacayo N., Brown P.O. (2012). Circular RNAs are the predominant transcript isoform from hundreds of human genes in diverse cell types. PLoS ONE.

[93] Jeck W.R., Sorrentino J.A., Wang K., Slevin M.K., Burd C.E., Liu J., Marzluff W.F., Sharpless N.E. (2013). Circular RNAs are abundant, conserved, and associated with ALU repeats. RNA.

[94] Memczak S., Jens M., Elefsinioti A., Torti F., Krueger J., Rybak A., Maier L., Mackowiak S.D., Gregersen L.H., Munschauer M. (2013). Circular RNAs are a large class of animal RNAs with regulatory potency. Nature.

[95] Hansen T.B., Jensen T.I., Clausen B.H., Bramsen J.B., Finsen B., Damgaard C.K., Kjems J. (2013). Natural RNA circles function as efficient microRNA sponges. Nature.

[96] Yan J., Yang Y., Fan X., Liang G., Wang Z., Li J., Wang L., Chen Y., Adetula A.A., Tang Y. (2022). circRNAome profiling reveals circFgfr2 regulates myogenesis and muscle regeneration via a feedback loop. J. Cachexia Sarcopenia Muscle.

[97] Zhang B., Chen Y., Chen Q., Zhang H. (2025). Exosomal miRNAs in muscle-bone crosstalk: Mechanistic links, exercise modulation and implications for sarcopenia, osteoporosis and osteosarcopenia. Metabolism.

[98] Yang Y., Hu G., Wan G., Li M., Chang B., Yi X. (2023). Idiopathic inflammatory myopathy and non-coding RNA. Front. Immunol..

[99] Nakamichi R., Ma S., Nonoyama T., Chiba T., Kurimoto R., Ohzono H., Olmer M., Shukunami C., Fuku N., Wang G. (2022). The mechanosensitive ion channel PIEZO1 is expressed in tendons and regulates physical performance. Sci. Transl. Med..

[100] Miyaki S., Sato T., Inoue A., Otsuki S., Ito Y., Yokoyama S., Kato Y., Takemoto F., Nakasa T., Yamashita S. (2010). MicroRNA-140 plays dual roles in both cartilage development and homeostasis. Genes. Dev..

[101] Zhang Y., Su S.A., Li W., Ma Y., Shen J., Wang Y., Shen Y., Chen J., Ji Y., Xie Y. (2021). Piezo1-Mediated Mechanotransduction Promotes Cardiac Hypertrophy by Impairing Calcium Homeostasis to Activate Calpain/Calcineurin Signaling. Hypertension.

[102] Qiu H., Fu Y., Guo Z., Zhang X., Wang X., Wu H. (2024). Dysregulated microRNAs and long non-coding RNAs associated with extracellular matrix stiffness. Exp. Cell Res..

[103] Li M., Zhang X., Wang M., Wang Y., Qian J., Xing X., Wang Z., You Y., Guo K., Chen J. (2022). Activation of Piezo1 contributes to matrix stiffness-induced angiogenesis in hepatocellular carcinoma. Cancer Commun..

[104] Chen X., Chen J. (2022). miR-10b-5p-mediated upregulation of PIEZO1 predicts poor prognosis and links to purine metabolism in breast cancer. Genomics.

[105] Zhang N., Wu P., Mu M., Niu C., Hu S. (2024). Exosomal circZNF800 Derived from Glioma Stem-like Cells Regulates Glioblastoma Tumorigenicity via the PIEZO1/Akt Axis. Mol. Neurobiol..

[106] Qin C., Feng Y., Yin Z., Wang C., Yin R., Li Y., Chen K., Tao T., Zhang K., Jiang Y. (2024). The PIEZO1/miR-155-5p/GDF6/SMAD2/3 signaling axis is involved in inducing the occurrence and progression of osteoarthritis under excessive mechanical stress. Cell Signal.

[107] Huang L., Li L., Chen X., Zhang H., Shi Z. (2016). MiR-103a targeting Piezo1 is involved in acute myocardial infarction through regulating endothelium function. Cardiol. J..

[108] Liu H., Zhou L., Yi P., Zhan F., Zhou L., Dong Y., Xiong Y., Hua F., Xu G. (2024). ω3-PUFA alleviates neuroinflammation by upregulating miR-107 targeting PIEZO1/NFκB p65. Int. Immunopharmacol..

[109] Wu T., Zhou Y., Jiang Y., Li X., Shi W., Wang Z., Guo S., Wang Y., Li T. (2025). A novel BMSC-derived exosomal circRNA promotes angiogenesis by targeting miR-34a-5p to regulate Piezo1. Stem Cell Res. Ther..

[110] Li J., Ma D., Zhang C., Zheng X., Hao R., Zuo B., Xiao F., Li Y., Liu Y., Duan Z. (2025). Targeting miR-337 mitigates disuse-induced bone loss. Cell Discov..

[111] McGeary S.E., Lin K.S., Shi C.Y., Pham T.M., Bisaria N., Kelley G.M., Bartel D.P. (2019). The biochemical basis of microRNA targeting efficacy. Science.

[112] Chen Y., Wang X. (2020). miRDB: An online database for prediction of functional microRNA targets. Nucleic Acids Res..

[113] Sticht C., De La Torre C., Parveen A., Gretz N. (2018). miRWalk: An online resource for prediction of microRNA binding sites. PLoS ONE.

[114] Murakami A., Masuda A., Hirakawa T., Nakanishi R., Hirano K., Tsuchiya M., Nonomura K., Ono Y., Murayama T., Hara Y. (2026). Baculovirus-mediated gene transfer enables functional expression of the PIEZO1 ion channel in isolated muscle satellite cells. J. Cell Sci..

[115] McPherron A.C., Lawler A.M., Lee S.J. (1997). Regulation of skeletal muscle mass in mice by a new TGF-beta superfamily member. Nature.

[116] Bodine S.C., Latres E., Baumhueter S., Lai V.K., Nunez L., Clarke B.A., Poueymirou W.T., Panaro F.J., Na E., Dharmarajan K. (2001). Identification of ubiquitin ligases required for skeletal muscle atrophy. Science.

[117] Sandri M., Sandri C., Gilbert A., Skurk C., Calabria E., Picard A., Walsh K., Schiaffino S., Lecker S.H., Goldberg A.L. (2004). Foxo transcription factors induce the atrophy-related ubiquitin ligase atrogin-1 and cause skeletal muscle atrophy. Cell.

[118] Egan B., Zierath J.R. (2013). Exercise metabolism and the molecular regulation of skeletal muscle adaptation. Cell Metab..

[119] Coletti C., Acosta G.F., Keslacy S., Coletti D. (2022). Exercise-mediated reinnervation of skeletal muscle in elderly people: An update. Eur. J. Transl. Myol..

[120] Chen W., You W., Valencak T.G., Shan T. (2022). Bidirectional roles of skeletal muscle fibro-adipogenic progenitors in homeostasis and disease. Ageing Res. Rev..

[121] Zhang X., Liu X., Li Q., Li C., Li X., Qian J., Li J., Li X. (2025). GsMTx-4 combined with exercise improves skeletal muscle structure and motor function in rats with spinal cord injury. PLoS ONE.

