# Supplementary material for: Piezo1 Mechanotransduction in Skeletal Muscle: Convergence with Noncoding RNA Regulation in Myogenesis, Regeneration, and Sarcopenia
Source: Int J Mol Sci. 2026 Aug 7;27(16):7084. doi: 10.3390/ijms27167084 (PMC13513523; doi:10.3390/ijms27167084)
Supplement: Supplementary file 1 [file ijms-27-07084-s001.zip › ijms-4457696-supplementary.pdf]

Supplementary Table S1. Three-tool prediction of PIEZO1 3′UTR-targeting microRNAs (TargetScan ∩ miRDB ∩ miRWalk).

Gene: PIEZO1 (Entrez 9780). Candidates ranked by an adjusted composite score combining per-tool evidence; “Triple-DB hit” marks the six microRNAs returned by all three tools. Context++ = TargetScan context++ score; TarPmiR = miRWalk probability; miRTarBase indicates prior experimental validation of the miRNA-PIEZO1 pair in any tissue. The list is hypothesis-generating; each candidate requires direct experimental validation.

Rank	miRNA	DB count	Triple-DB hit	TargetScan seed family	TargetScan context++	TargetScan site type	TargetScan conservation	miRDB score	miRWalk TarPmiR	miRWalk best region	Composite score (adj.)	miRTarBase ID	miRTarBase validated
1	hsa-miR-139-5p	3	Yes	miR-139-5p	-0.59	8mer	Broadly conserved	82	0.92	3UTR	0.783		No
2	hsa-miR-101-3p	3	Yes	miR-101-3p	-0.56	8mer	Broadly conserved	81	0.85	3UTR	0.616		No
3	hsa-miR-16-5p	3	Yes	miR-15/16/195/424/497	-0.38	7mer-m8+8mer	Broadly conserved	72	0.92	CDS	0.569		No
4	hsa-miR-497-5p	3	Yes	miR-15/16/195/424/497	-0.38	7mer-m8+8mer	Broadly conserved	69	0.92	CDS	0.546		No
5	hsa-miR-15b-5p	3	Yes	miR-15/16/195/424/497	-0.38	7mer-m8+8mer	Broadly conserved	72	0.85	3UTR	0.429		No
6	hsa-miR-424-5p	3	Yes	miR-15/16/195/424/497	-0.38	7mer-m8+8mer	Broadly conserved	69	0.85	CDS	0.406		No
7	hsa-miR-103a-3p	2	No	miR-103-3p/107	-0.39	7mer-m8	Broadly conserved		0.92	CDS	0.601		No
8	hsa-miR-15a-5p	2	No	miR-15/16/195/424/497	-0.38	7mer-m8+8mer	Broadly conserved	72			0.591		No
9	hsa-miR-195-5p	2	No	miR-15/16/195/424/497	-0.38	7mer-m8+8mer	Broadly conserved	72			0.591		No
10	hsa-miR-320a-5p	2	No					56	1	CDS	0.532		No
11	hsa-miR-219a-5p	2	No					77	0.92	3UTR	0.531		No
12	hsa-miR-34a-5p	2	No	miR-34-5p/449-5p	-0.06	7mer-m8	Broadly conserved		1	3UTR	0.512		No
13	hsa-miR-7112-3p	2	No					50	1	3UTR	0.462		No
14	hsa-miR-184	2	No	miR-184	-0.41	7mer-1A	Broadly conserved		0.85	CDS	0.406		No
15	hsa-miR-4632-3p	2	No					56	0.92	CDS	0.286		No
16	hsa-miR-3191-3p	2	No					52	0.92	CDS	0.239	MIRT490026	Yes
17	hsa-miR-24-3p	2	No	miR-24-3p	-0.19	7mer-m8	Broadly conserved		0.85	CDS	0.182		No
18	hsa-miR-144-3p	2	No	miR-144-3p	-0.16	7mer-1A	Broadly conserved	53			0.177		No
19	hsa-let-7c-5p	1	No						1	CDS	1		No
20	hsa-let-7e-5p	1	No						1	CDS	1		No
21	hsa-miR-100-5p	1	No						1	CDS	1		No
22	hsa-miR-106a-5p	1	No						1	CDS	1		No
23	hsa-miR-10a-5p	1	No						1	CDS	1		No
24	hsa-miR-139-3p	1	No						1	CDS	1		No
25	hsa-miR-17-5p	1	No						1	CDS	1		No
26	hsa-miR-21-3p	1	No						1	CDS	1		No
27	hsa-miR-27a-5p	1	No						1	CDS	1		No
28	hsa-miR-30a-3p	1	No						1	CDS	1		No
29	hsa-miR-31-5p	1	No						1	CDS	1		No
30	hsa-miR-568	1	No					96			1		No
31	hsa-miR-96-3p	1	No						1	CDS	1		No
32	hsa-miR-99a-5p	1	No						1	CDS	1		No
33	hsa-miR-3912-5p	1	No					91			0.891		No
34	hsa-miR-107	1	No	miR-103-3p/107	-0.39	7mer-m8	Broadly conserved				0.673		No
35	hsa-miR-4782-3p	1	No					77			0.587		No
36	hsa-miR-6766-3p	1	No					77			0.587		No
37	hsa-miR-3127-5p	1	No	miR-3127-5p	-0.33	7mer-m8	Poorly conserved				0.509		No
38	hsa-miR-6838-5p	1	No					72			0.478		No
39	hsa-let-7a-5p	1	No						0.92	CDS	0.467		No
40	hsa-miR-93-5p	1	No						0.92	CDS	0.467		No
41	hsa-miR-96-5p	1	No						0.92	CDS	0.467		No

Rank	miRNA	DB count	Triple-DB hit	TargetScan seed family	TargetScan context++	TargetScan site type	TargetScan conservation	miRDB score	miRWalk TarPmiR	miRWalk best region	Composite score (adj.)	miRTarBase ID	miRTarBase validated
42	hsa-miR-5692a	1	No					64			0.304		No
43	hsa-miR-491-5p	1	No	miR-491-5p	-0.21	7mer-m8	Conserved (Mammals)				0.303		No
44	hsa-miR-3605-5p	1	No	miR-3605-5p	-0.22	7mer-m8	Poorly conserved				0.302		No
45	hsa-miR-5010-5p	1	No	miR-5010-5p	-0.22	7mer-1A	Poorly conserved				0.302		No
46	hsa-miR-642a-5p	1	No	miR-642a-5p	-0.2	8mer	Poorly conserved				0.264		No
47	hsa-miR-153-5p	1	No					62			0.261		No
48	hsa-miR-371a-3p	1	No	miR-371a-3p	-0.19	7mer-m8	Poorly conserved				0.245		No
49	hsa-miR-625-5p	1	No	miR-625-5p	-0.19	7mer-m8	Poorly conserved				0.245		No
50	hsa-miR-3918	1	No	miR-3918	-0.16	7mer-m8	Poorly conserved				0.189		No
51	hsa-miR-3150b-3p	1	No	miR-3150b-3p	-0.14	7mer-m8	Poorly conserved				0.151		No
52	hsa-miR-588	1	No	miR-588	-0.14	7mer-m8	Poorly conserved				0.151		No
53	hsa-miR-449a	1	No	miR-34-5p/449-5p	-0.06	7mer-m8	Broadly conserved				0.05		No
54	hsa-miR-214-3p	1	No	miR-214-3p/3619-5p	-0.08	7mer-m8	Poorly conserved				0.038		No
55	hsa-miR-3619-5p	1	No	miR-214-3p/3619-5p	-0.08	7mer-m8	Poorly conserved				0.038		No
56	hsa-miR-942-5p	1	No	miR-942-5p	-0.07	7mer-m8	Poorly conserved				0.019		No
57	hsa-miR-10b-5p	1	No						0.85	CDS	0	MIRT006294	Yes

Supplementary Table S2. Skeletal-muscle abundance and tissue specificity of predicted and literature PIEZO1-interacting microRNAs versus canonical myomiRs.

RPMM (reads per million mapped) = mean of four human skeletal-muscle donor samples from miRNATissueAtlas2 (GEO GSE163534). Organ TSI (tissue-specificity index, 0-1; 1 = perfectly organ-specific) and peak organ/system are from the miRNATissueAtlas2 organ/system-specificity resource. None of the predicted or literature candidates peaks in skeletal muscle, whereas all five canonical myomiRs peak in muscle with organ TSI 0.94-0.99. The predicted candidates are expressed in muscle but not muscle-enriched, and require the direct experimental tests of Prediction 2.

miRNA	group	skeletal-muscle RPMM (mean)	peak organ	organ tissue-specificity index (TSI)	peak organ system	muscle-enriched?
hsa-miR-139-5p	Prediction-2 candidate (Triple-DB)	489	spleen	0.764	nervous	No
hsa-miR-101-3p	Prediction-2 candidate (Triple-DB)	5374	spleen	0.381	lymphatic	No
hsa-miR-16-5p	Prediction-2 candidate (Triple-DB)	4518	bone	0.628	lymphatic	No
hsa-miR-497-5p	Prediction-2 candidate (Triple-DB)	299	artery	0.533	cardiovascular	No
hsa-miR-15b-5p	Prediction-2 candidate (Triple-DB)	342	bone	0.666	lymphatic	No
hsa-miR-424-5p	Prediction-2 candidate (Triple-DB)	916	testis	0.865	urinary	No
hsa-miR-214-3p	Table 4 literature interaction		bladder	0.506	respiratory	No
hsa-miR-103a-3p	Table 4 literature interaction		vein	0.58	nervous	No
hsa-miR-107	Table 4 literature interaction		kidney	0.618	lymphatic	No
hsa-miR-34a-5p	Table 4 literature interaction		testis	0.587	cardiovascular	No
hsa-miR-10b-5p	Table 4 literature interaction		kidney	0.867	urinary	No
hsa-miR-155-5p	Table 4 literature interaction		spleen	0.842	lymphatic	No
hsa-miR-625-5p	Table 4 literature interaction		nerve	0.699	nervous	No
hsa-miR-337-3p	Table 4 literature interaction		testis	0.562	urinary	No
hsa-miR-582-5p	Table 4 literature interaction		bone	0.642	nervous	No
hsa-miR-1-3p	canonical myomiR	185637	muscle	0.977	musculoskeletal	Yes
hsa-miR-133a-3p	canonical myomiR	295770	muscle	0.98	musculoskeletal	Yes
hsa-miR-133b	canonical myomiR	51558	muscle	0.979	musculoskeletal	Yes
hsa-miR-206	canonical myomiR	36872	muscle	0.988	musculoskeletal	Yes
hsa-miR-499a-5p	canonical myomiR	2716	muscle	0.944	musculoskeletal	Yes
